# T‐2 Toxin‐Mediated β‐Arrestin‐1 O‐GlcNAcylation Exacerbates Glomerular Podocyte Injury via Regulating Histone Acetylation

**DOI:** 10.1002/advs.202307648

**Published:** 2023-12-11

**Authors:** Tushuai Li, Wenxue Sun, Shenglong Zhu, Chengsheng He, Tong Chang, Jie Zhang, Yongquan Chen

**Affiliations:** ^1^ School of Biology and Food Engineering Changshu Institute of Technology Suzhou 215500 P.R. China; ^2^ Wuxi School of Medicine Jiangnan University Wuxi 214013 P.R. China; ^3^ Wuxi Translational Medicine Research Center and Jiangsu Translational Medicine Research Institute Wuxi Branch Wuxi 214013 P.R. China; ^4^ Translational Pharmaceutical Laboratory Jining First People's Hospital Shandong First Medical University Jining 272000 P.R. China; ^5^ Postdoctoral of Shandong University of Traditional Chinese Medicine Ji'nan 250355 P.R. China; ^6^ Institute of Translational Pharmacy Jining Medical Research Academy Jining 272000 P.R. China

**Keywords:** β‐arrestin‐1, glomerular injury, histone acetylation, o‐glcnacylation, podocyte injury, T‐2 toxins

## Abstract

T‐2 toxin causes renal dysfunction with proteinuria and glomerular podocyte damage. This work explores the role of metabolic disorder/reprogramming‐mediated epigenetic modification in the progression of T‐2 toxin‐stimulated podocyte injury. A metabolomics experiment is performed to assess metabolic responses to T‐2 toxin infection in human podocytes. Roles of protein O‐linked‐N‐acetylglucosaminylation (O‐GlcNAcylation) in regulating T‐2 toxin‐stimulated podocyte injury in mouse and podocyte models are assessed. O‐GlcNAc target proteins are recognized by mass spectrometry and co‐immunoprecipitation experiments. Moreover, histone acetylation and autophagy levels are measured. T‐2 toxin infection upregulates glucose transporter type 1 (GLUT1) expression and enhances hexosamine biosynthetic pathway in glomerular podocytes, resulting in a significant increase in β‐arrestin‐1 O‐GlcNAcylation. Decreasing β‐arrestin‐1 or O‐GlcNAc transferase (OGT) effectively prevents T‐2 toxin‐induced renal dysfunction and podocyte injury. Mechanistically, O‐GlcNAcylation of β‐arrestin‐1 stabilizes β‐arrestin‐1 to activate the mammalian target of rapamycin (mTOR) pathway as well as to inhibit autophagy during podocyte injury by promoting H4K16 acetylation. To sum up, OGT‐mediated β‐arrestin‐1 O‐GlcNAcylation is a vital regulator in the development of T‐2 toxin‐stimulated podocyte injury via activating the mTOR pathway to suppress autophagy. Targeting β‐arrestin‐1 or OGT can be a potential therapy for T‐2 toxin infection‐associated glomerular injury, especially podocyte injury.

## Graphical abstract

T‐2 toxin exposure promotes β‐arrestin‐1 O‐linked‐N‐acetylglucosaminylation by the glucose transporter type 1‐dependent hexosamine biosynthesis pathway. Modification of β‐arrestin‐1 with O‐linked N‐acetylglucosamine at Thr98 stabilizes β‐arrestin‐1 by preventing ubiquitin‐dependent proteolysis. It stabilizes β‐arrestin‐1 to suppress autophagy via activating the mammalian target of rapamycin pathway through recruitment of p300 to upregulate H4K16 acetylation in T‐2 toxin‐resistant glomerular podocytes.

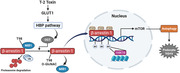

## 1 Introduction

As a sesquiterpenoid fungal metabolite, T‐2 toxin is a trichothecene mycotoxin (type A subgroup). It is the most toxic and common one and is produced by different fusarium species from various crops such as corn, wheat, and barley.^[^ ^1^ ^]^ A survey of 8721 agricultural products in 75 countries in 2018 by Biomin Company shows that the T‐2 toxin pollution rate is as high as 23% and the average detection amount is 25 mg k^−1^g, which is much higher than other pollutants.^[^ ^2^ ^]^ The high detection rate and wide distribution of T‐2 toxin have posed an underlying threat to human health.^[^ ^3^ ^]^ Chronic exposure to T‐2 toxin is associated with renal dysfunction in mice, accompanied by proteinuria, podocyte vacuolation, and foot process abnormality.^[^ ^4^ ^]^

Podocytes are highly specialized and ultimately differentiated glomerular epithelial cells and serve as indispensable parts of the glomerular ultrafiltration system to prevent proteinuria.^[^ ^5^ ^]^ Podocytes have an intrinsic system to withstand stresses and will undergo injury if the stresses exceed their compensatory capacity. Podocyte injury is characterized by foot process effacement, blebs, cytoplasmic vacuoles, and abnormalities in organelles and the cell membrane.^[^ ^6^ ^]^ Podocyte injury leads to complex biological responses, which have significant roles in maintaining the function of the glomerulus. The complex glomerular responses are actually wound‐healing processes aimed at suppressing the impacts of podocyte injury.^[^ ^7^ ^]^ The filtration barrier function is stabilized by a tight network composed of cell adhesion molecules, actin cytoskeleton, and slit membrane molecules. Defects of this network in injured podocytes cause proteinuria.^[^ ^8^ ^]^ Previous studies using gene disruption techniques have confirmed that each molecular event occurring during podocyte injury serves as a potential target for podocyte‐directed therapies.^[^ ^7^ ^]^ There have been no studies on T‐2 toxin infection‐induced podocyte injury, and this study aimed to elucidate the underlying molecular mechanisms of T‐2 toxin‐triggered glomerular podocyte injury.

β‐arrestins belong to multifunctional proteins, the negative regulators of G protein‐coupled receptors (GPCRs).^[^ ^9^ ^]^ They act as molecular scaffolds that interact with intracellular partner proteins to regulate multiple physiological and pathological processes including immune response, tumorigenesis, and inflammation.^[^ ^10^ ^]^ The β‐arrestins family includes 4 subunit proteins, of which arrestins 1 and 4 are solely expressed in the cones and rods of the retina, and arrestins 2 and 3 (β‐arrestin‐1 and β‐arrestin‐2) are ubiquitously distributed in tissues of mammals.^[^ ^11^ ^]^ Numerous literature have demonstrated the capacity of β‐arrestin‐2 to modulate addiction‐associated behaviors and microglia chemotaxis in rodents via activating the extracellular receptor kinase pathway and antiviral immune response.^[^ ^12^ ^]^ Furthermore, β‐arrestin‐1 disorder induces podocyte injury in mouse models of adriamycin‐induced nephropathy and streptozotocin‐induced diabetic nephropathy through activation of β‐catenin in a Wnt‐independent or independent pathway.^[^ ^13^ ^]^ Interestingly, in addition to acting as a protein scaffold that regulates cellular function, β‐arrestin‐1 has also been found to exert an intranuclear function that mediates histone acetylation by recruiting and interacting with histone acetyltransferase p300.^[^ ^14^ ^]^ However, whether dysregulation of β‐arrestin‐1 participates in T‐2 toxin‐induced podocyte damage remains unknown.

Cellular metabolic disorder/reprogramming is an important factor causing tissue cell damage by mediating epigenetic regulation such as histone modification, microRNA (miRNA) degradation, and post‐translational modification (PTM).^[^ ^15^ ^]^ As a type of PTM, O‐linked β‐N‐acetylglucosamine modification (O‐GlcNAcylation) refers to that O‐GlcNAc transferase (OGT) catalyzes the addition of N‐acetylglucosamine (GlcNAc) to serine or threonine residues of target protein.^[^ ^16^ ^]^ Uridine diphosphate N‐acetylglucosamine (UDP‐GlcNAc) belongs to one of the most important substrate donors of O‐GlcNAcylation, which is derived from the hexosamine biosynthesis pathway (HBP), a branch of glucose metabolism. Normally, ≈2%–5% of the total glucose inside cells is transformed into UDP‐GlcNAc, which is essential for maintaining cellular activity and homeostasis.^[^ ^17^ ^]^ However, in the case of large amounts of glucose accumulations in metabolically disordered cells, the excessive generation of UDP‐GlcNAc may provide adequate donors for HBP‐mediated O‐GlcNAcylation, resulting in protein aberrant O‐GlcNAcylation with unpredictable consequences. Recent literature has certified the crucial role of protein O‐GlcNAcylation in kidney diseases.^[^ ^18^ ^]^ O‐GlcNAcylation promotes the internalization of megalin in the proximal tubule and increases stabilization of serine‐threonine kinase, resulting in proteinuria and renal fibrosis in mouse models of diabetic nephropathy^[^ ^19^ ^]^ and unilateral ureteral obstruction.^[^ ^20^ ^]^ Although OGT‐mediated O‐GlcNAcylation has been revealed to be indispensable for the maturation and survival of the podocyte foot process,^[^ ^21^ ^]^ the potential of disordered protein O‐GlcNAcylation in T‐2 toxin‐induced podocyte damage remains unclear.

In this paper, we probed metabolic responses of mice glomeruli and human podocytes to T‐2 toxin infection. T‐2 toxin infection‐induced metabolic reprogramming of glucose metabolism toward the HBP pathway along with enhanced β‐arrestin‐1 O‐GlcNAc modification in mouse glomeruli and podocytes in vitro. O‐GlcNAcylation of β‐arrestin‐1 accelerated renal dysfunction and podocyte damage in T‐2 toxin‐fed mice. Functionally, β‐arrestin‐1 modified by O‐GlcNAc group blocked the interaction of β‐arrestin‐1 with E3 ubiquitin ligase mind bomb 1 (MIB1) to resist ubiquitin‐dependent proteolysis, thereby promoting H4K16 acetylation to activate mammalian target of rapamycin (mTOR) pathway and inhibit autophagy during podocyte injury. Our results collectively indicate that intervening in OGT‐mediated β‐arrestin‐1 O‐GlcNAcylation can be a potential therapeutic strategy in T‐2 toxin‐stimulated podocyte injury.

## 2 Results

### 2.1 T‐2 Toxin Infection Upregulates GLUT1 Expression and Enhances β‐arrestin‐1 O‐GlcNAcylation in Mouse Glomeruli and Human Podocytes

**Figure 1:**
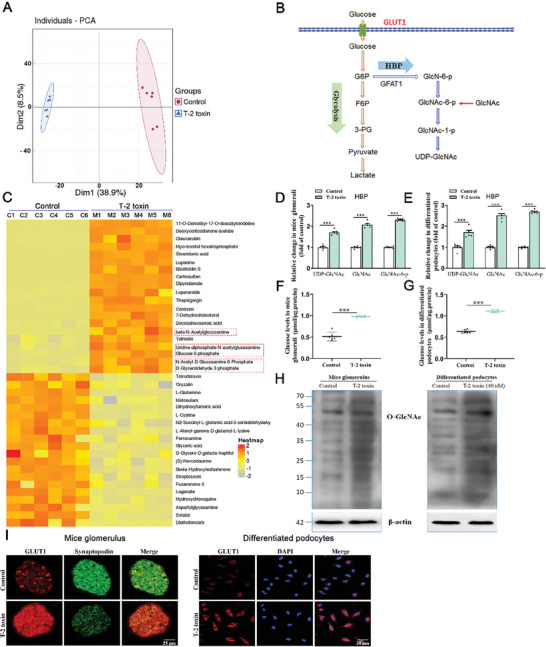
T‐2 toxin infection facilitates HBP as well as elevates protein O‐GlcNAcylation. A) Metabolomics experiment was used to detect principal components of podocytes infected with or without T‐2 toxin for 24 h (*n =* 6). B) An overview of the HBP. C) Heatmap of differential metabolites under the control or T‐2 toxin‐infected conditions. D,E) Relative alterations in intermediate metabolites of HBP in mouse glomeruli D) and podocytes E) (*n =* 6). F–G) Relative alterations in the levels of glucose in mouse glomeruli F) and podocytes G) (*n =* 6). H) Western blot analysis of total O‐GlcNAc modified protein from mouse glomeruli (left pan) and podocytes (right pan). I) Immunofluorescence staining analysis of GLUT1 (red) in mouse glomeruli (left pan) and podocytes (right pan) (*n =* 3). The scale bar is 25 µm (left panel) and 30 µm (right panel). ^***^ *p <* 0.001; data are expressed as mean ± SEM; unpaired t test was used.

To probe the metabolic changes after T‐2 toxin infection, a metabolomics analysis was conducted in podocytes (24 h) treated with or without T‐2 toxin (40 nM). Principal component analysis displayed that T‐2 toxin infection obviously altered the intracellular metabolic profile of podocytes (**Figure** **1**A). Recent literature has revealed that metabolic reprogramming, particularly altered glucose metabolism, has a key function in podocyte injury.^[^ ^15^, ^22^ ^]^ Hence, we explored the impact of glucose metabolism in T‐2 toxin‐exposed podocytes. The expression levels of D‐glyceraldehyde 3‐phosphate, beta‐N‐Acetylglucosamine, N‐Acetyl‐D‐Glucosamine 6‐phosphate, GlcNAc‐6‐P, and UDP‐GlcNAc (the end‐product of HBP) in glucose metabolism followed by T‐2 toxin infection were elevated (Figure 1B,C). ELISA results showed that UDP‐GlcNAc, GlcNAc, and GLCNAC‐6‐P expression levels were higher in the glomeruli of T‐2‐toxin‐fed mice than in controls (Figure 1D). These results were similar in T‐2 toxin‐exposed podocytes (Figure 1E). Glucose levels were also elevated in T‐2 toxin group in both in vivo (Figure 1F) and in vitro models (Figure 1G). Because HBP enhancement usually leads to increased protein O‐GlcNAcylated modification, we examined whether T‐2 toxin infection can affect O‐GlcNAc modification in podocytes. It was found that protein O‐GlcNAcylation was significantly increased in mouse glomeruli 28 days post T‐2 toxin infection (Figure 1H, left panel). A similar result was observed in T‐2 toxin‐infected podocytes (24 h) (Figure 1H, right panel). GLUT1 is a main glucose transporter localized on the cell membrane, and OGT is an active enzyme responsible for catalyzing protein O‐GlcNAcylation.^[^ ^16^ ^]^ We next detected GLUT1 and OGT protein levels in T‐2 toxin‐fed mouse glomeruli and T‐2 toxin‐exposed podocytes. As shown in Figure 1I and Figure S1 (Supporting Information), GLUT1 protein levels were increased in T‐2 toxin‐fed mouse glomeruli, as well as in T‐2 toxin‐cultured podocytes. Nevertheless, we did not discover obvious alterations in OGT protein levels of these animal and cell models. These findings suggested that T‐2 toxin infection upregulates GLUT1 expression, facilitates glucose uptake, and elevates UDP‐GlcNAc synthesis and protein O‐GlcNAcylation in glomerular podocytes.

**Figure 2:**
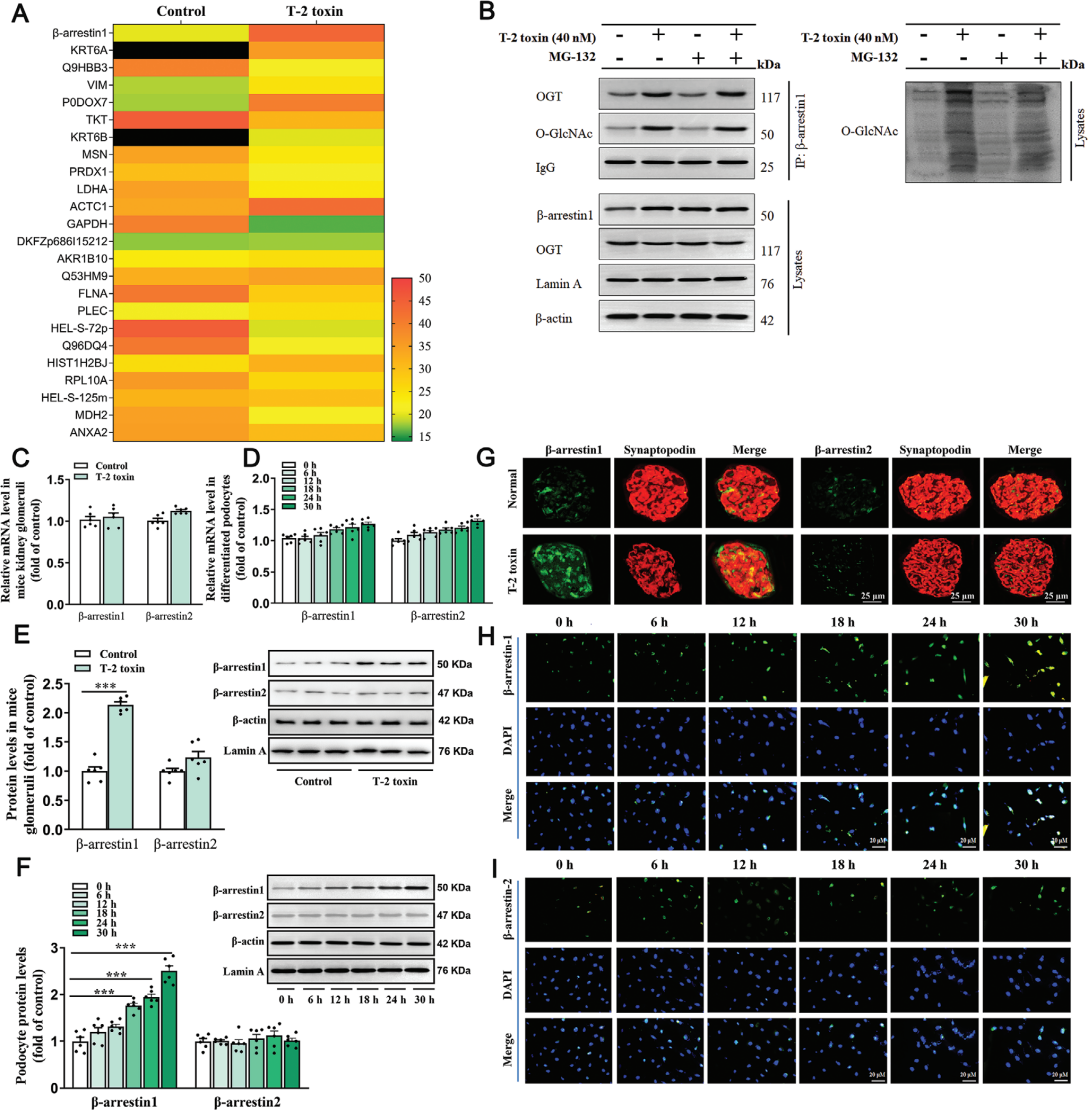
T‐2 toxin infection increases β‐arrestin‐1 O‐GlcNAcylation and enhances β‐arrestin‐1 stability in glomerular podocytes. A) O‐GlcNAc modified proteins were analyzed by Co‐IP combined with LC‐MS, and some target proteins have higher scores, as shown in figure A. B) Endogenous interplays of β‐arrestin‐1 with OGT and O‐GlcNAc. T‐2 toxin‐exposed podocytes treated with or without MG‐132 (2.5 µM) were subjected to CoIP using β‐arrestin‐1 antibody, followed by Western blot analysis. C,D) qPCR quantification analysis of β‐arrestin‐1 along with β‐arrestin‐2 mRNA levels in mouse glomeruli C) and podocytes D) (*n =* 6). E,F) Western blot analysis of β‐arrestin‐1 and β‐arrestin‐2 protein levels in mouse glomeruli E) and podocytes F) (*n =* 6). G–I) Immunofluorescence staining analysis of β‐arrestin‐1 and β‐arrestin‐2 in mouse glomeruli G) and in podocytes H,I) (*n =* 3). ^***^ *p <* 0.001; data are expressed as mean ± SEM; unpaired t‐test was used in 2C, 2E, and one‐way ANOVA followed by Dunnett's post hoc test was used in 2D and 2F.

To further clarify the role of O‐GlcNAcylation in glomerular podocytes, the putative O‐GlcNAc‐modified proteins in T‐2 toxin‐exposed podocytes were screened using immunoprecipitation mass spectrometry (IP‐MS) analysis. A total of 36 candidate O‐GlcNAc‐modified proteins were recognized, and β‐arrestin‐1 was the most significant one in T‐2 toxin‐exposed podocytes (**Figure** **2**A). We next focused on β‐arrestin‐1, which possesses a crucial potential in G protein‐coupled receptor signaling.^[^ ^9a^ ^]^ Interplays between OGT and β‐arrestin‐1 were displayed by a co‐immunoprecipitation (Co‐IP) assay in T‐2 toxin‐stimulated podocytes. More importantly, T‐2 toxin exposure dramatically increased the O‐GlcNAcylation levels of β‐arrestin‐1 in podocytes (Figure 2B). These findings revealed that β‐arrestin‐1 can be O‐GlcNAcylated by OGT after T‐2 toxin infection in glomerular podocytes.

### 2.2 β‐arrestin‐1 Overexpression Exacerbates Podocyte Injury Upon T‐2 Toxin Treatment

To elucidate the potential of β‐arrestin‐1 dysfunction underlying podocyte homeostasis and injury, we first examined the mRNA and protein levels of β‐arrestin‐1 and its subunit β‐arrestin‐2 in mouse glomeruli and podocytes upon T2 exposure. It was discovered that both β‐arrestin‐1 and β‐arrestin‐2 mRNA levels did not change in the mouse (Figure 2C) and cell models (Figure 2D). However, β‐arrestin‐1 protein levels were elevated in T‐2 toxin‐fed mouse glomeruli (Figure 2E) and in podocytes following T‐2 toxin exposure (Figure 2F). The immunofluorescence staining results confirmed the enhanced β‐arrestin‐1 protein expression in T‐2 toxin‐fed mouse glomeruli (Figure 2G) and in podocytes following T‐2 toxin exposure (Figure 2H). No obvious changes were found in β‐arrestin‐2 protein levels in T‐2 toxin‐stimulated podocytes (Figure 2I).

**Figure 3:**
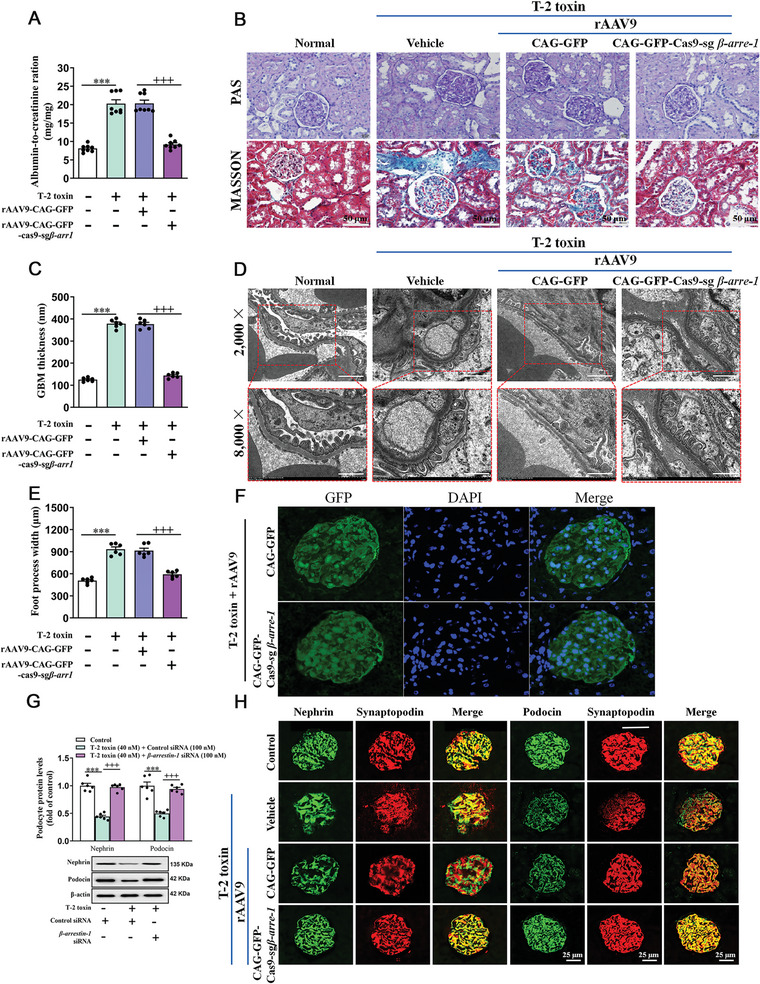
β‐arrestin‐1 overexpression exacerbates podocyte injury upon T‐2 toxin exposure. A) The urine albumin‐to‐creatinine ratio in T‐2 toxin‐fed mice treated with rAAV‐CAG‐GFP‐Cas9‐sg*β‐arrestin‐1* or rAAV‐CAG‐GFP (*n =* 6). B) Representative images of PAS as well as MASSON‐stained renal cortex sections were exhibited (scale bar, 50 µm) (*n =* 3). C) GBM thickness. D) Number of foot processes of mice under transmission electron microscope (TEM) (Scale bar, 2 µm in upper panel; 500 nm in lower panel, *n =* 5). E) Foot process width. F) The infection efficiency of rAAV‐CAG‐GFP‐Cas9‐sg*β‐arrestin‐1* or rAAV‐CAG‐GFP in mice glomeruli was validated by immunofluorescence staining (*n =* 3, scale bar, 20 µm). G) Quantitation of Nephrin as well as Podocin protein levels in podocytes after transfection of control siRNA or *β‐arrestin‐1* siRNA and their corresponding controls by western blot (*n =* 6). H) Immunofluorescence staining was used to analyze Nephrin and Podocin in mouse glomeruli infected with CAG‐GFP‐Cas9‐sg*β‐arrestin‐1* or CAG‐GFP (*n =* 3, scale bar, 25 µm). ^***^ *p <* 0.001, ^+++^ *p <* 0.001, data are expressed as mean ± SEM; one‐way ANOVA followed by Tukey's post hoc test was used.

Furthermore, we evaluated renal dysfunction and podocyte injury in T‐2 toxin‐treated mice. Consistent with a previous study,^[^ ^4^ ^]^ T‐2 toxin exposure markedly increased albumin‐to‐creatinine ratio (**Figure** **3**A), glomerular glycogen and collagen deposition (Figure 3B) caused glomerular hypertrophy (Figure 3B), as well as increased glomerular basement membrane (GBM) thickness in mice (Figure 3C). Glomerular podocyte injury was also induced by T‐2 toxin, manifested by podocyte isolation from GBM with partial effacement of podocyte foot process (Figure 3D,E). Next, β‐arrestin‐1 knockout mice were constructed by recombinant adeno‐associated virus (rAAV9) in this study, and rAAV9 exhibits an excellent transfection efficiency in mouse glomeruli (Figure 3F). Figure S1C (Supporting Information) revealed that rAAV9 CAG‐GFP‐Cas9‐sg*β‐arrestin‐1* successfully decreased β‐arrestin‐1 expression in mouse kidneys. After 28 days of T‐2 toxin feeding, β‐arrestin‐1 knockout mice developed a lower albumin‐to‐creatinine ratio (Figure 3A), glomerular glycogen and collagen deposition (Figure 3B), and GBM thickness than T‐2 toxin‐fed normal mice (Figure 3C). Meanwhile, β‐arrestin‐1 deletion prevented T‐2 toxin‐caused podocyte foot process thickening and effacement (Figure 3C–D), foot process width enlargement (Figure 3E) along with the reduction on expression of Nephrin as well as Podocin (Figure 3G–I). These findings revealed that β‐arrestin‐1 overexpression is induced by T‐2 toxin and participates in renal dysfunction and podocyte damage.

### 2.3 β‐arrestin‐1 Mediates Histone H4 Hyperacetylation Via Recruiting p300 in T‐2 Toxin‐Induced Podocyte Injury

**Figure 4:**
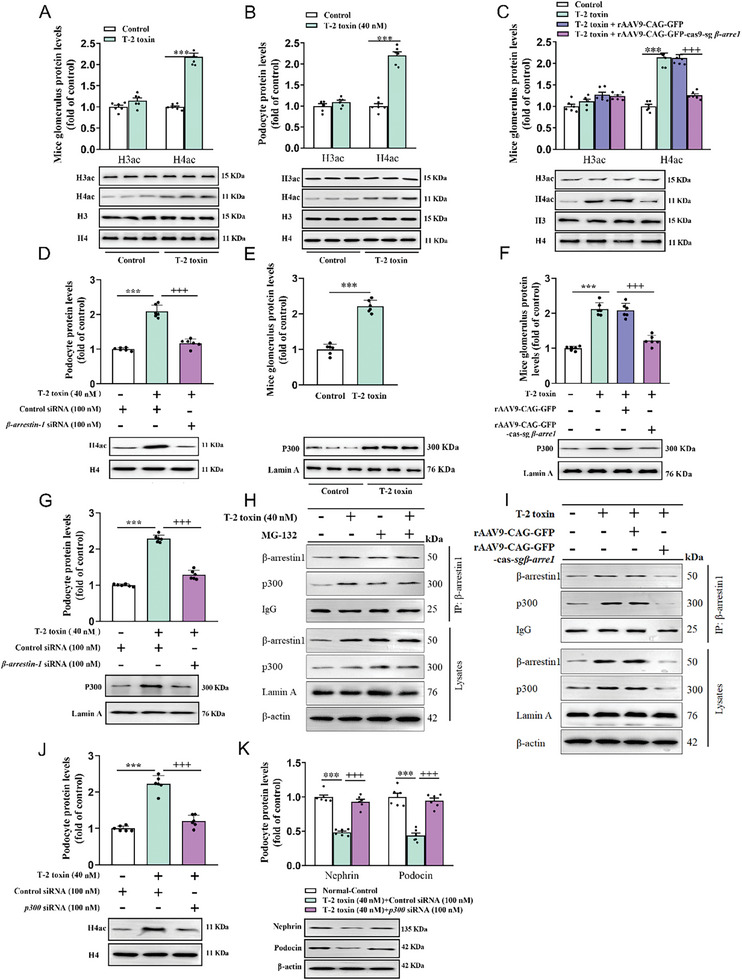
β‐arrestin‐1 mediates histone H4 hyperacetylation via recruiting p300 in T‐2 toxin‐induced podocyte injury. (A‐B) Western blot analyzed H3ac and H4ac protein levels in glomeruli of T‐2 toxin‐ stimulated mice A) and in T‐2 toxin‐treated podocytes B) (*n =* 6). C) Western blot analysis of H3ac and H4ac protein levels in glomeruli of mice transfected with CAG‐GFP‐Cas9‐sg*β‐arrestin‐1* or CAG‐GFP (*n =* 6). D) Western blot analysis of H4ac protein levels in podocytes transfected with control siRNA or *β‐arrestin‐1* siRNA (*n =* 6). E) Western blot analyzed p300 protein levels in glomeruli (*n =* 6). F) Western blot analysis of p300 protein levels in glomeruli of mice transfected with CAG‐GFP‐Cas9‐sg*β‐arrestin‐1* or CAG‐GFP (*n =* 6). G) Western blot analysis of p300 protein levels in podocytes transfected with*β‐arrestin‐1* siRNA (*n =* 6). H) Podocytes were treated with or without T‐2 toxin in the presence or absence of MG‐132. The interaction of β‐arrestin‐1 and p300 was determined by Co‐IP combined with Western blot analysis. I) Co‐IP combined with Western blot was used to detect β‐arrestin‐1 and p300 protein interaction in glomeruli of mice transfected with CAG‐GFP‐Cas9‐sg*β‐arrestin‐1* or CAG‐GFP. J,K) Western blot analysis of J) H4ac, K) Nephrin and Podocin protein levels in podocytes after transfection with *p300* siRNA (*n =* 6). ^*^ *p <* 0.05, ^+++^ *p <* 0.001, data are expressed as mean ± SEM. Unpaired t‐test was used in 4A, 4B, and 4E, and one‐way ANOVA followed by Tukey's post hoc test was used in 4C, 4D, 4F, 4G, 4J, and 4K.

β‐arrestin‐1 has been validated to modulate histone acetylation and gene transcription by recruiting p300.^[^ ^14^, ^23^ ^]^ Therefore, we first analyzed histone acetylation levels and p300 nuclear accumulation in mouse glomeruli and podocytes upon T‐2 toxin exposure. Acetylation modification of histone 4 was enhanced in the animal and cellular models as compared to normal controls, while histone 3 acetylation levels were unchanged (**Figure** **4**A,B). However, knockout of β‐arrestin‐1 with rAAV9 was able to reverse T‐2 toxin‐induced increase of histone 4 acetylation levels in mouse glomeruli (Figure 4C). These results were further confirmed in T‐2 toxin‐infected podocytes with *β‐arrestin‐1* siRNA transfection (Figure 4D), suggesting that β‐arrestin‐1 overexpression may participate in histone 4 acetylation in response to T‐2 toxin exposure. We also found that p300 nuclear accumulation was significantly increased in mouse glomeruli and podocytes after T‐2 toxin infection (Figure 4E,F). Furthermore, decreasing β‐arrestin‐1 by rAAV9 or siRNA obviously blocked p300 nuclear accumulation in T‐2 toxin‐infected mice and cells (Figure 4F,G).

Next, to investigate whether β‐arrestin‐1 exacerbates H4 acetylation and podocyte injury by recruiting p300, we measured the interaction of β‐arrestin‐1 with p300. As shown in Figure 4H,I, Co‐IP results clearly confirmed the binding activity of β‐arrestin‐1 and p300 in mouse glomeruli and podocytes upon T‐2 toxin infection, while β‐arrestin‐1 deficiency significantly inhibited their binding. Furthermore, the knockdown of p300 also prevented T‐2 toxin‐induced histone 4 acetylation modification and downregulation of Nephrin and Podocin expression in podocytes (Figure 4J,K). A rescue assay was performed using both p300 siRNA and β‐arrestin‐1 siRNA. The results revealed that β‐arrestin‐1 siRNA restored the promotive effects of p300 siRNA on Nephrin and Podocin protein expression (Figure S2A, Supporting Information). These findings demonstrate that β‐arrestin‐1 binds to p300 protein to mediate histone H4 hyperacetylation T‐2 toxin‐stimulated podocytes.

### 2.4 β‐arrestin‐1 Inhibits Autophagy to Exacerbate Podocyte Damage Through Acetylation of H4K16 to Activate mTOR Signaling Pathway

**Figure 5:**
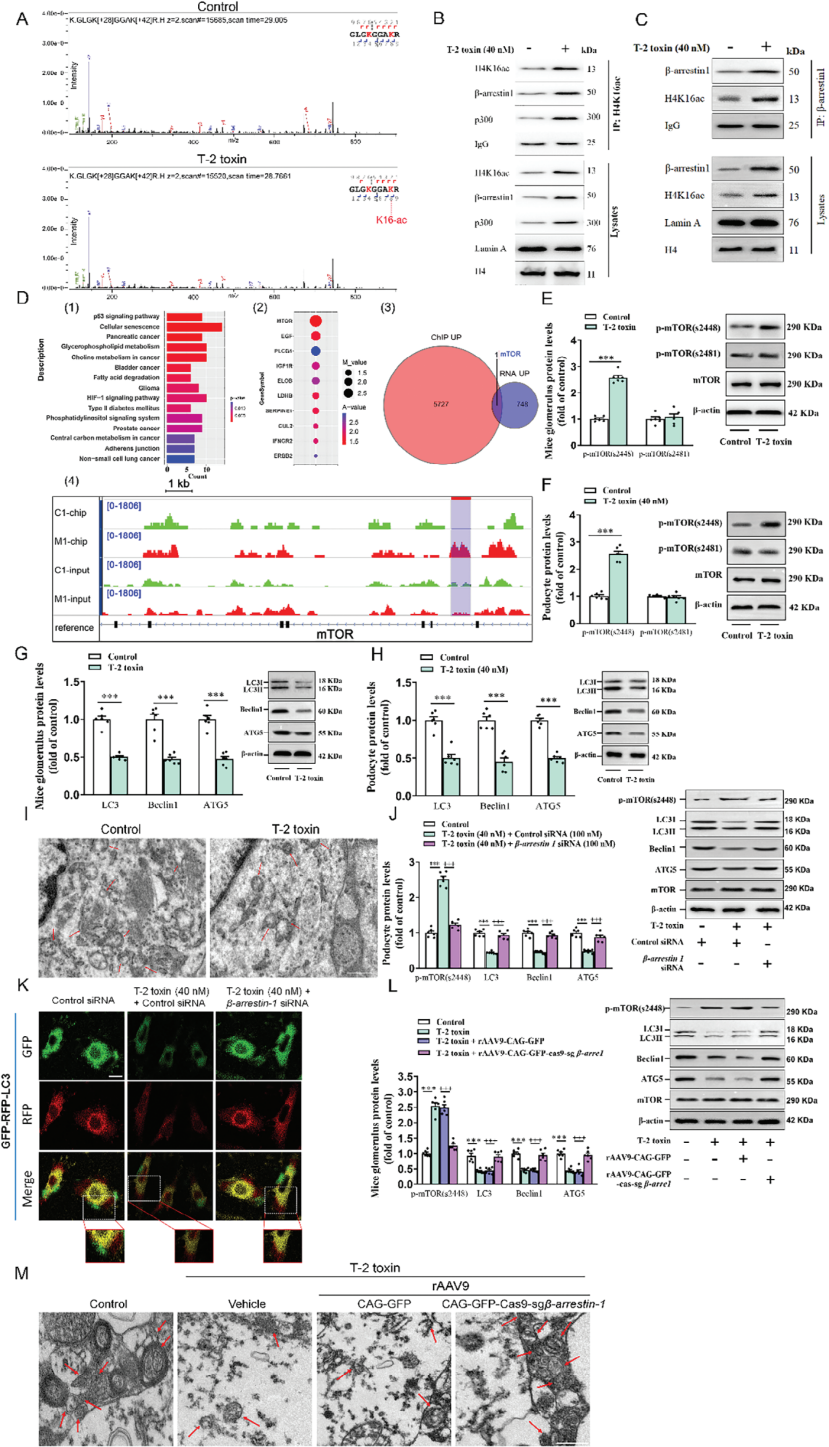
β‐arrestin‐1 inhibits autophagy to exacerbate podocyte damage through acetylation of H4K16 to activate mTOR pathway. A) H4 acetylation at lysine 16 was detected by LC‐MS/MS in T‐2 toxin‐exposed podocytes. (B‐C) Endogenous interactions of H4K16 with β‐arrestin‐1 and p300 in podocytes were assessed using Co‐IP assay with H4K16 B) or β‐arrestin‐1 C) antibodies. D) Chromatin immunoprecipitation sequencing (ChIP‐Seq) analysis of H4K16 targeted binding gene fragments in T‐2 toxin‐exposed podocytes. 1) Kyoto Encyclopedia of Genes and Genomes (KEGG) pathway analysis of ChIP‐Seq results showed the activation of mTOR signaling pathway in T‐2 toxin‐exposed podocytes. 2) The bubble diagram showed that H4K16ac is significantly enriched in the mTOR promoter region. 3) The Venn map showed that mTOR promoter is the only overlapping candidate gene by ChIP‐Seq and RNA‐Seq results. 4) Normalized ChIP‐seq tracks showed the enrichment of H4K16ac on mTOR promoter. Regions with differences in H4K16ac enrichment were highlighted in the lavender box. (E‐H) Western blot analysis of p‐mTOR (s2448) (E,F), p‐mTOR (s2481) (E,F), light chain 3 (LC3) (G,H), Beclin1 (G,H), and autophagy related 5 (ATG5) (G‐H) protein levels in mouse glomeruli (E and G) and podocytes (F and H) (*n =* 6). I) Representative electronic micrographs showed the number of autophagosomes in glomerular podocytes (*n =* 3). The arrows exhibit autophagosomes. Scale bar: 1 µm. J) Western blot analysis of p‐mTOR (s2448), LC3, Beclin1, and ATG5 in podocytes after transfection with *β‐arrestin‐1* siRNA. K) LC3 staining in podocytes (*n =* 3). Scale bar, 20 µm. L) Western blot analysis of p‐mTOR (s2448), LC3, Beclin1, and ATG5 in glomeruli of mice infected with rAAV‐CAG‐GFP‐Cas9‐sg*β‐arrestin‐1* or rAAV‐CAG‐GFP (*n =* 6). M) The number of autophagosomes in mice glomerular podocytes was shown by TEM (*n =* 3). ^***^ *p <* 0.001, ^+++^ *p <* 0.001, data are expressed as mean ± SEM; unpaired t‐test was used in 5E, 5F, 5G, and 5H, and one‐way ANOVA followed by Tukey's post hoc test was used in 5J and 5L.

We further analyzed acetylation sites on histone 4 using MS through IP of histone 4 in T‐2 toxin‐treated podocytes. As shown in **Figure** **5**A, the H4K16 site was mostly bound by acetyl group in podocytes, which is consistent with a previous report in TGF‐β1‐stimulated mouse lung fibroblasts with marked histone H4K16 acetylation in the NOX4 promoter region.^[^ ^24^ ^]^ MS results were further confirmed in T‐2 toxin‐exposed podocytes by Western blot (Figure 5B,C). H4K16 acetylation enhancement is related to transcriptional activation.^[^ ^25^ ^]^ Specifically, excessive acetyl group binding at the H4K16 site leads to the loose binding of H4K16 to its interacting DNA, which is beneficial for transcription factors to initiate the transcriptional expression of target genes.

To identify specific factors or signaling pathways involved in H4K16ac‐mediated podocyte injury, we then performed a ChIP‐Seq analysis to investigate the differentially enriched genes that bound to H4K16ac in T‐2 toxin‐stimulated podocytes. Kyoto Encyclopedia of Genes and Genomes (KEGG) analysis displayed that p53 signaling pathway and cellular senescence were enriched in T‐2 toxin‐exposed podocytes. The mTOR gene fragment showed the most abundant binding with H4K16ac and was the only gene identified by both ChIP‐Seq and RNA‐Seq (Figure 5D). KEGG and heatmap analysis of RNA‐Seq results showed that the mTOR pathway was significantly enriched in mouse glomeruli post‐28 days of T‐2 toxin infection versus the normal controls, accompanied by significant inhibition of autophagy (Figure S2, Supporting Information). These results indicate that the mTOR signaling pathway may take part in T‐2 toxin‐stimulated podocyte injury.

It has been reported that mTOR is a key regulatory switch for autophagy and usually leads to autophagy inhibition.^[^ ^26^ ^]^ We subsequently examined the phosphorylation states of mTOR and the autophagy‐related proteins LC3, Beclin1, and ATG5 (three widely used markers of autophagy) to determine whether β‐arrestin‐1 regulates podocyte autophagy by activating mTOR. We discovered that T‐2 toxin exposure significantly increased cellular p‐mTOR (Ser2448) protein levels (Figure 5E,F), decreased LC3 II/I, Beclin1, and ATG5 (Figure 5G,H) protein levels, and did not influence p‐mTOR (Ser2481) levels (Figure 5E,F) in T‐2 toxin‐infected glomeruli and podocytes. TEM analysis further revealed that T‐2 toxin markedly decreased the number of typical double‐membrane autophagosomes in mouse glomerular podocytes (Figure 5I). We also constructed a tandem RFP‐GFP‐LC3 adenovirus to verify the induction of autophagy by morphological points representing autophagosome formation in T‐2 toxin‐stimulated podocytes. Decreasing β‐arrestin‐1 by siRNA significantly decreased phosphorylation of mTOR at Ser2448 and upregulated LC3 II/I, Beclin1, as well as ATG5 protein levels (Figure 5J). In Figure 5K, T‐2 toxin induced less red puncta, while knocking down β‐arrestin‐1 obviously restored fluorescence intensity in T‐2 toxin‐exposed podocytes. Decreasing β‐arrestin‐1 by rAAV9 also upregulated LC3 II/I, Beclin1, ATG5 protein levels and decreased p‐mTOR protein levels (Figure 5L), promoted the formation of autophagosomes in T‐2 toxin‐infected glomeruli (Figure 5M). The above data demonstrate that β‐arrestin‐1 overexpression activates the mTOR pathway to inhibit autophagy through upregulation of H4K16 acetylation. To further determine whether p300 participates in the H4K16 hyperacetylation‐mediated activation of mTOR pathway and inhibition of podocyte autophagy, we next transfected LV5‐p300 into T‐2 toxin‐exposed podocytes following transfection of *β‐arrestin‐1* siRNA. As a result, overexpression of p300 increased mTOR phosphorylation at Ser2448, decreased LC3 II/I, Beclin1, and ATG5 protein levels, and suppressed autophagosome formation (Figure S3A,B, Supporting Information).

Taken together, these data prove that T‐2 toxin increases β‐arrestin‐1 to promote H4K16 hyperacetylation by recruiting p300, and then activates the mTOR pathway to inhibit autophagy in podocytes.

### 2.5 Modification of β‐arrestin‐1 with O‐linked N‐acetylglucosamine Stabilizes β‐arrestin‐1 by Preventing Ubiquitin‐Dependent Proteolysis

**Figure 6:**
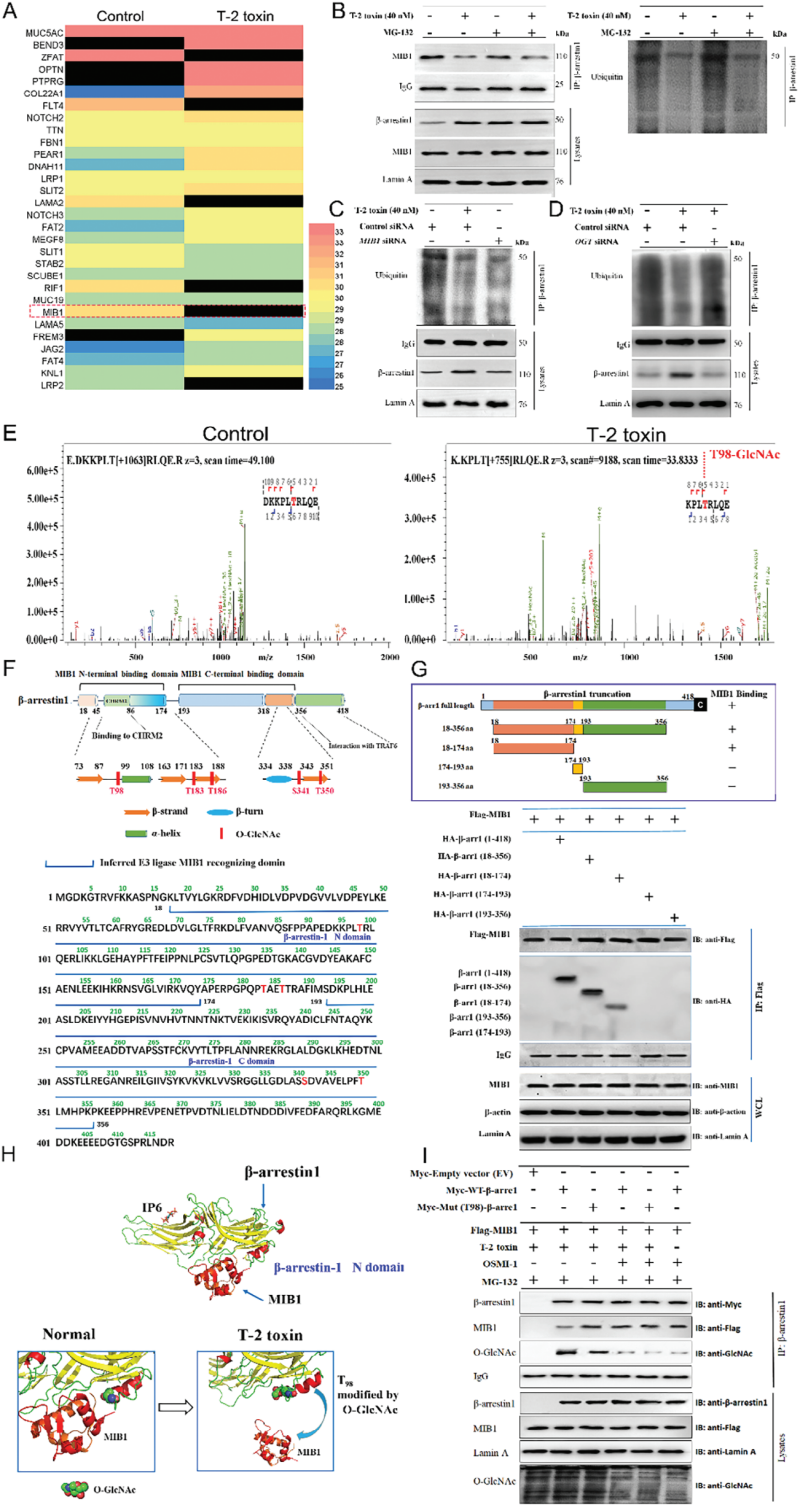
Modification of β‐arrestin‐1 with O‐linked N‐acetylglucosamine stabilizes β‐arrestin‐1 by preventing its ubiquitin‐dependent proteolysis. A) Expression of β‐arrestin‐1 binding proteins in normal or T‐2 toxin‐exposed podocytes was shown in a heat map. B) Endogenous interplays of β‐arrestin‐1 with MIB1 and ubiquitin. T‐2 toxin‐exposed podocytes with or without MG‐132 were subject to Co‐IP using a β‐arrestin‐1 antibody, followed by Western blot analysis with MIB1 and ubiquitin antibodies. (C‐D) Co‐IP and Western blot analyzed β‐arrestin‐1 ubiquitination levels in podocytes cultured with or without T‐2 toxin transfected with control siRNA, *MIB1* siRNA C) or *OGT* siRNA D). E) GlcNAc‐modified β‐arrestin‐1 at site threonine 98 was detected by LC‐MS/MS in T‐2 toxin‐exposed podocytes as compared with normal controls. F) Five candidate O‐GlcNAcylated residues of β‐arrestin‐1, identified by MS, were exhibited in the context of protein domains, secondary structure elements, along with interacting partners of β‐arrestin‐1. The figure below shows the predictive β‐arrestin‐1 domain for MIB1 binding. G) Co‐IP combined with Western blot analysis of the MIB1 binding domain in β‐arrestin‐1 in podocytes transfected with Flag‐tagged MIB1 overexpressed plasmid or different HA‐tagged β‐arrestin‐1 truncation plasmids. H) Molecular docking using PyMol showed that the O‐GlcNAcylation of β‐arrestin‐1 at sites of threonine 98 leads to the dissociation of MIB1 from β‐arrestin‐1. I) Myc‐tagged β‐arrestin‐1 constructs and Flag‐tagged MIB1 were transfected into Crispr/Cas9 *β‐arrestin‐1*‐KO podocytes, and then incubated with T‐2 toxin and MG‐132 in the presence or absence of OSMI‐1, and anti‐β‐arrestin‐1 antibody was used for IP. Precipitates were analyzed by Western blot analysis with indicated antibodies.

Because β‐arrestin‐1 overgeneration only occurs at the protein levels without affecting its genome transcription, β‐arrestin‐1 may only undergo PTMs in glomerular podocytes upon T‐2 toxin exposure. O‐GlcNAcylation stabilizes substrate proteins against E3 ubiquitin ligase‐mediated ubiquitin‐dependent proteolysis.^[^ ^27^ ^]^ Therefore, we hypothesized that OGT‐mediated addition of β‐N‐acetylglucosamine (GlcNAc) may block β‐arrestin‐1 against degradation by the ubiquitin‐proteasome system by competing with E3 ubiquitin ligase for β‐arrestin‐1 binding. To verify this, first, protein lysates were immunoprecipitated with anti‐β‐arrestin‐1 antibodies from podocytes, and then subjected to LC‐MS analysis for the identification of β‐arrestin‐1‐binding proteins. The results exhibited that only 6 β‐arrestin‐1‐binding proteins were significantly down‐regulated in T‐2 toxin‐exposed podocytes. Among them, only MIB1 is an E3 ubiquitin ligase (**Figure** **6**A). Next, we discovered that T‐2 toxin reduced the interplay of β‐arrestin‐1 and MIB1 as well as the ubiquitination level of MIB1 (Figure 6B). In the following experiments, we detected the degree of β‐arrestin‐1 ubiquitination by knocking down MIB1 in normal podocytes and decreasing OGT in T‐2 toxin‐exposed podocytes via siRNA. As expected, knockdown of MIB1 significantly reduced β‐arrestin‐1 ubiquitination in normal podocytes, while decreasing OGT reversed T‐2 toxin‐induced down‐regulation in β‐arrestin‐1 ubiquitination levels (Figure 6C,D), suggesting that T‐2 toxin may prevent β‐arrestin‐1 from interacting with MIB1 to inhibit β‐arrestin‐1 ubiquitin‐dependent degradation through triggering β‐arrestin‐1 O‐GlcNAc modification. As exhibited in Figure S4A–D (Supporting Information) and Figure 6E, five serine/threonine sites of β‐arrestin‐1 in T‐2 toxin‐exposed podocytes were confirmed to be significantly O‐GlcNAcylated by LC‐MS/MS. Among them, one putative O‐GlcNAc site (Thr98) was located in the N‐terminal binding domain, which was able to bind to MIB1. Two putative O‐GlcNAc sites (Thr183 and Thr186) were located between the N‐terminal and C‐terminal binding domain, while 2 additional potential O‐GlcNAc sites (Ser341 and Thr350) were located in the C‐terminal of MIB1binding domains (Figure 6F). Interestingly, the view network predicted that β‐arrestin‐1 contains two MIB binding domains, an N‐terminal binding domain (18‐174 aa), and a C‐terminal binding domain (193‐356 aa) (Figure 6F). To further elucidate the mechanism of O‐GlcNAcylation in stabilizing β‐arrestin‐1 in T‐2 toxin‐treated podocytes, recombinant β‐arrestin‐1 was purified from podocytes to perform the site‐mapping experiments. We subsequently constructed the β‐arrestin‐1 full‐length plasmid along with four β‐arrestin‐1 deletion plasmids. The Co‐IP results revealed that the N‐terminal binding domain of β‐arrestin‐1 is required for its interplay with MIB1 (Figure 6G). Additionally, molecular docking analysis showed that the O‐GlcNAcylation of β‐arrestin‐1 at sites threonine 98caused the dissociation of MIB1 from β‐arrestin‐1, while the other four sites did not (Figure 6H). Furthermore, we created a non‐glycosylated mutant plasmid in the threonine 98 of β‐arrestin‐1 by mutating threonine to alanine and constructed Crispr/Cas9 β‐arrestin‐1‐knockout podocytes to examine whether individual unglycosylatable β‐arrestin‐1 mutant lost its capacity to facilitate the dissociation of MIB1 from β‐arrestin‐1 under T‐2 toxin condition. As a result, mutation of β‐arrestin‐1 at threonine 98 significantly increased the binding of MIB1to β‐arrestin‐1 and decreased O‐GlcNAc modification. After inhibiting O‐GlcNAc modification using OGT inhibitor OSMI‐1, wild‐type β‐arrestin‐1 displayed enhanced binding capacity to MIB1 accompanied by a reduction in its O‐GlcNAc modification (Figure 6I). These results reveal that O‐GlcNAcylation of β‐arrestin‐1 at Thr98 induced by T‐2 toxin is sufficient to inhibit its binding to MIB1 and stabilizes β‐arrestin‐1 by preventing its ubiquitin‐dependent proteolysis in glomerular podocytes.

### 2.6 OGT Deletion Reduces β‐arrestin‐1 Expression and Alleviates mTOR Pathway‐Mediated Glomerular Podocyte Injury in T‐2 Toxin‐fed Mice

**Figure 7:**
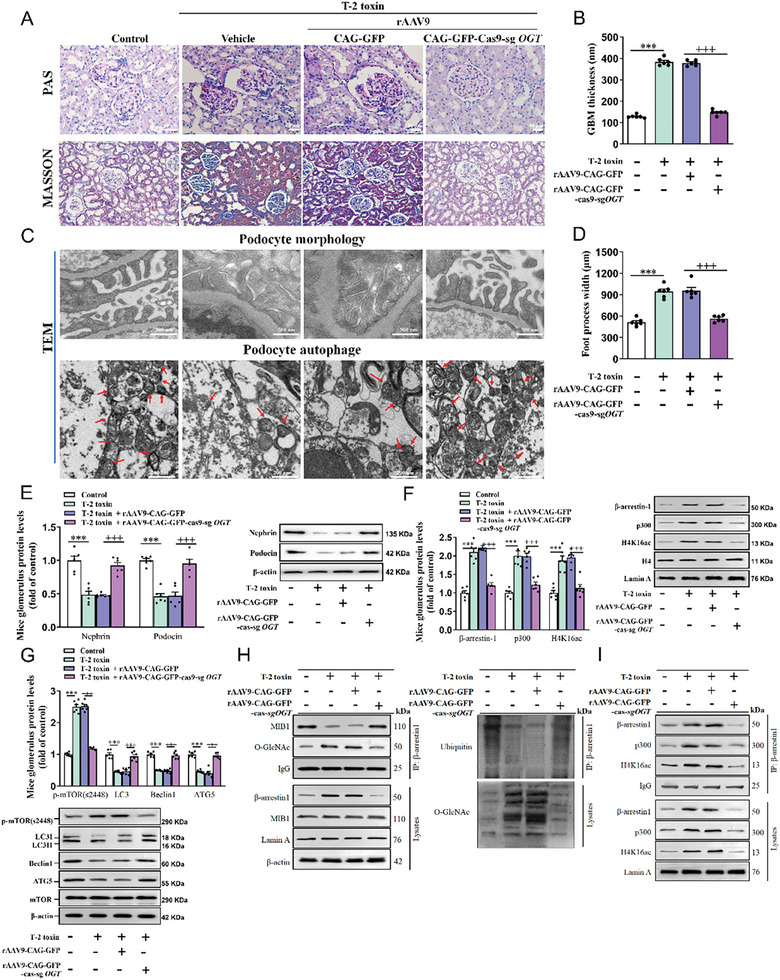
OGT deletion reduces β‐arrestin‐1 expression and alleviates mTOR pathway‐mediated glomerular podocyte injury in T‐2 toxin‐fed mice. A) Representative images of PAS and MASSON‐stained renal cortex sections (scale bar, 40 µm, *n =* 3). B) GBM thickness. C) TEM showed podocyte morphology (Scale bar, 500 nm, *n =* 5) and number of autophagosomes (Scale bar, 200 nm, *n =* 5). D) Foot process width. (E‐G) Western blot analyzed Nephrin E), Podocin E), β‐arrestin‐1 F), p300 F), H4K16ac F), p‐mTOR (s2448) G), LC3 G), Beclin1 G) and ATG5 G) protein levels in glomeruli (*n =* 6). H) The interplays of β‐arrestin‐1 with MIB1, O‐GlcNAc, and β‐arrestin‐1 ubiquitination and O‐GlcNAcylation in isolated glomeruli were analyzed by Co‐IP. I) The interaction of β‐arrestin‐1 with p300 and H4K16ac in isolated glomeruli was analyzed by Co‐IP. ^***^ *p <* 0.001, ^+++^ *p <* 0.001, data are expressed as mean ± SEM; one‐way ANOVA followed by Tukey's post hoc test was used.

We also generated OGT knockout mice to clarify the role of β‐arrestin‐1 O‐GlcNAcylation in T‐2 toxin‐induced glomerular podocyte injury. Immunofluorescence and Western blot confirmed an excellent transfection and knockout efficiency of rAAV9 in mouse kidneys (Figure S5A,B, Supporting Information). In T‐2 toxin‐fed mice, knockout of OGT markedly reduced albumin‐to‐creatinine ratio (Figure S5C, Supporting Information), glomerular glycogen and collagen deposition (**Figure** **7**A), GBM thickening (Figure 7B), alleviated foot process effacement, suppressed formation of autophagosome (Figure 7C), decreased podocyte foot process width, and increased Nephrin and Podocin expression (Figure 7D‐E). Consistently, OGT deficiency also significantly down‐regulated β‐arrestin‐1 and p300 nuclear accumulation, decreased H4K16 acetylation modification (Figure 7F) and p‐mTOR (s2448) protein levels with inhibition of LC3 II/I, Beclin1, and ATG5 protein expression (Figure 7G) in glomeruli of T‐2 toxin‐fed mice. p62 is an autophagy adaptor protein and accumulates when autophagy is inhibited.^[^ ^28^ ^]^ We also detected p62 protein expression in both in vivo and in vitro models. As revealed in Figure S6A,B (Supporting Information), β‐arrestin‐1 inhibition caused a significant decrease in p62 protein expression in T‐2 toxin‐stimulated in vitro podocytes and *ex vivo* glomeruli. Knockout of OGT by rAAV9 also leads to the reduction of p62 protein in glomeruli of T‐2 toxin‐stimulated mice (Figure S6C, Supporting Information). p300 rescued the effects of β‐arrestin‐1 siRNA on p62 protein expression (Figure S6D, Supporting Information). Subsequently, OGT deletion by rAAV9‐Cas9 *sgOGT* promoted the binding of β‐arrestin‐1 to MIB1 and β‐arrestin‐1 ubiquitin‐dependent proteolysis and abolished β‐arrestin‐1 O‐GlcNAcylation modification in glomeruli (Figure 7H). Furthermore, OGT deficiency reduced the interplay of β‐arrestin‐1 with p300 and H4K16 proteins (Figure 7I). These findings confirmed that T‐2 toxin stabilizes β‐arrestin‐1 through enhancement of β‐arrestin‐1 O‐GlcNAcylation to elevate H4K16 acetylation via recruiting p300, thus facilitating the phosphorylation of mTOR at Ser2448 to suppress autophagy and cause podocyte injury.

## 3 Discussion

Although previous literature has revealed that T‐2 toxin infection induces renal dysfunction accompanied by proteinuria and glomerular podocyte ultrastructural abnormalities, the potential and possible mechanisms of T‐2 toxin‐triggered podocyte damage remain obscure. In this research, we identified the alterations of major intracellular metabolic profiles and signaling pathways associated with T‐2 toxin‐induced podocyte epigenetic modification via a metabolomics assay combined with IP‐MS, RNA‐Seq, and ChIP‐Seq. Our results demonstrated that T‐2 toxin increased glomerular podocyte GLUT1 expression, thus promoting glucose uptake in glomerular podocytes. The elevated nutrient state consequently offered sufficient substrates for the HBP to produce UDP‐GlcNAc, resulting in elevated protein O‐GlcNAcylation. Notably, we discovered that β‐arrestin‐1 O‐GlcNAcylated modification was associated with T‐2 toxin‐induced glomerular podocyte damage. Mechanistically, we revealed that OGT‐mediated O‐GlcNAcylation stabilized β‐arrestin‐1 to mediate glomerular podocyte injury through upregulation of histone acetylation to active the mTOR signaling pathway.

Increasingly studies demonstrated that β‐arrestins disorders are linked to podocyte injury in a variety of glomerular diseases. β‐arrestin‐2 mediates Nephrin endocytosis along with injured slit diaphragm integrity through interaction with the Nephrin C terminus in streptozotocin‐induced hyperglycemia mice.^[^ ^29^ ^]^ As a molecular scaffold, β‐arrestin‐1 recruits CXCR4 and Src to generate a CXCR4/β‐arrestin‐1/Src signaling body, inducing podocyte injury in an adriamycin‐induced mouse model of nephropathy.^[^ ^13b^ ^]^ Herein, we determined that β‐arrestin‐1, rather than β‐arrestin‐2, mediated T‐2 toxin‐induced glomerular podocyte injury. Whereas inconsistent with previous reports that β‐arrestin‐1 works as a cytosolic regulator and scaffold, we observed that β‐arrestin‐1 mainly accumulated in the nucleus of podocytes upon T‐2 toxin infection in both mouse and cell models. Considering the nuclear function of β‐arrestin‐1 in modulating histone acetylation and gene transcription, we studied β‐arrestin‐1‐mediated histone modification and found that over‐accumulation of β‐arrestin‐1 in nucleus inhibited autophagy to exacerbate podocyte damage through upregulation of histone H4 acetylation to activate the mTOR signaling pathway. Our results suggested a novel GPCRs signaling‐independent pathway of β‐arrestin‐1 nuclear function and revealed that β‐arrestin‐1‐mediated the T‐2 toxin‐induced podocyte damage via regulation of histone acetylation.

The present study also clarified the mechanism of how β‐arrestin‐1 activated the mTOR pathway in the inhibition of podocyte autophagy in a histone acetylation manner. As a scaffold protein, β‐arrestin‐1 was reported to recruit p300, a histone acetyltransferase, to enhance local histone H4 acetylation and transcription of downstream genes, which was validated in β‐arrestin‐1 knockout HEK293, HeLa, and murine embryonic fibroblast (MEF) cells.^[^ ^14^ ^]^ We used IP‐MS analysis and functional verification in podocytes with or without T‐2 toxin exposure and displayed that β‐arrestin‐1 regulated mTOR activation by recruiting p300 to add acetyl group to the H4K16 site. There are some studies proving that β‐arrestin‐1 exerts intranuclear regulatory functions involving histone modifications in a p300‐dependent pathway. For example, β‐arrestin‐1 binds to p300 to form a β‐arrestin‐1/p300/Sp1 signalosome, promoting human telomerase reverse transcriptase transcription by triggering histone H4 acetylation in leukemia‐initiating cells of B‐lineage acute lymphoblastic leukemia.^[^ ^23^ ^]^ β‐arrestin‐1 works as a transcription co‐activator that combines β‐catenin and TFC4 to promote histone acetylation and chromatin reorganization on target genes containing ET‐1 by recruiting p300 in oxaliplatin (OX) and 5‐fluorouracil‐treated CRC stem‐like cells.^[^ ^30^ ^]^ In the present research, we have shown that p300‐mediated histone acetylation at the H4K16 locus represents a new perspective in revealing the mechanism of β‐arrestin‐1‐induced podocyte injury through epigenetic modification. Specifically, β‐arrestin‐1 activated mTOR expression to repress autophagy via binding with p300, and subsequently upregulated histone H4K16 acetylation in T‐2 toxin‐induced podocyte injury. Emerging evidence has indicated that podocytes maintain a high level of basal autophagy to ensure cell viability and homeostasis, and reduced autophagic activity is implicated in the progression of podocyte injury‐associated kidney diseases.^[^ ^31^ ^]^ Our evidence from β‐arrestin‐1 knockout mice and human podocytes suggested that β‐arrestin‐1 overgeneration blocked autophagosome formation in T‐2 toxin‐induced glomerular podocyte damage. β‐arrestin‐1/2 was reported to negatively regulate podocyte autophagy by intervening in ATG12‐ATG5 conjugation in streptozotocin‐induced podocyte dysfunction in diabetic mice.^[^ ^32^ ^]^ In contrast to this study that β‐arrestin‐1 exerts cytoplasmic modulatory function, we propose a novel mechanism that β‐arrestin‐1 interacted with p300 mediates histone H4K16 acetylation to activate mTOR signaling pathway, resulting in the inhibition of podocyte autophagy. It is essential to further explain the molecular mechanism underlying β‐arrestin‐1‐mediated autophagy suppression in podocyte injury under different pathological conditions.

Similar to metabolic reprogramming in tumorigenesis, podocyte metabolic reorganization is accompanied by cytoskeletal rearrangement, cellular vacuolization, and loss of functional proteins after stimulation by small‐molecule metabolites or xenobiotics. Excessive dietary fructose drives mitochondria metabolic remodeling via upregulation of ketone body generation, especially β‐hydroxybutyrate, to elevate overall acetylation levels of histone H3 and H4, participates in mitochondrial dysfunction, and ultimately leads to podocyte injury in a rat model.^[^ ^15a^ ^]^ Podocyte metabolic reorganization by complete pharmacologic repression of mTOR shifts the cellular energy metabolism toward lessening oxidative phosphorylation and anaerobic glycolysis, finally triggering reactive oxygen species production to exacerbate glomerulosclerosis in a mouse model of adriamycin nephropathy.^[^ ^33^ ^]^ In this study, we identified that T‐2 toxin caused GLUT1 high expression on the podocyte surface, excessive glucose intake in podocytes, and abnormal activation of HBP, indicting a metabolic reorganization in glomerular podocytes response to T‐2 toxin exposure. In fact, excess glucose intake may be metabolically shunted to HBP to produce UDP‐GlcNAc, which provides sufficient substrate for O‐GlcNAcylation of target proteins in these podocytes. Furthermore, our results revealed that O‐GlcNAcylation was responsible for high β‐arrestin‐1 nuclear accumulation, and this event was the starting point for T‐2 toxin‐induced glomerular podocyte injury. Our study built a bridge linking metabolic reorganization to histone epigenetic regulation in glomerular podocytes upon T‐2 toxin exposure.

It was reported that hyper‐O‐GlcNAcylation can stabilize substrate protein by resisting E3 ubiquitin‐mediated proteolysis in tumorigenesis, immune metabolic diseases, and diabetes and its complications. OGT‐mediated protein O‐GlcNAcylation causes the inhibition of a nuclear ubiquitin‐proteasome system, which is responsible for serum response factor stabilization and Krüppel‐like factor‐4 repression in vascular smooth muscle differentiation in aneurysm and injury‐induced neointimal hyperplasia.^[^ ^34^ ^]^ Our study illustrated that T‐2 toxin stimulation reduced β‐arrestin‐1 ubiquitination and its binding with MIB1 in podocytes and glomeruli of mice. Of note, β‐arrestin‐1 was significantly modified with O‐GlcNAc at five different amino acid sites (Thr98, Thr183, Thr186, Ser341, and Thr350) in T‐2 toxin‐exposed podocytes. Interestingly, among the five amino acid positions, Thr98, Ser341, and Thr350 are located in the predicted MIB1 binding domain. Further functional analysis indicated that β‐arrestin‐1 O‐GlcNAcylation at Thr98 avoided its productive interaction with MIB1 in podocytes under T‐2 toxin exposure. Many studies have demonstrated that O‐GlcNAc competes with binding substrate protein serine/threonine to regulate protein stability. In response to extracellular glucose stimuli, DOT1L is O‐GlcNAcylated at evolutionarily conserved Ser1511 in its C terminus, which stabilizes DOT1L and activates the downstream target genes by inhibiting the interaction of DOT1L with its E3 ubiquitin ligase UBE3C, ultimately promoting cell proliferation of leukemia in a xenograft nude mouse model.^[^ ^35^ ^]^ O‐GlcNAcylation of sine oculis homeobox homolog 1 at position 276 (Thr276) blocks the ubiquitin‐dependent proteolysis to enhance its stability in a xenograft mouse model of hepatocellular carcinoma.^[^ ^27b^ ^]^ In contrast, in vesicular stomatitis virus‐stimulated mice, OGT‐mediated O‐GlcNAcylation of mitochondrial antiviral signaling (MAVS) protein at Ser 366 site is responsible for MAVS K63‐linked ubiquitination and the antiviral signaling of gene‐like receptors.^[^ ^36^ ^]^ In our study, the strongest functionally relevant O‐GlcNAcylation site Thr98 existed in the MIB1 domain of β‐arrestin‐1 N‐terminal binding and prevented β‐arrestin‐1 from being degraded by ubiquitin‐dependent proteasome. Our loss‐of‐function assays illustrated that mutation of β‐arrestin‐1 at Thr98 abolished the dissociation of β‐arrestin‐1 from MIB1 in T‐2 toxin‐exposed podocytes. Therefore, the O‐GlcNAcylation mediated nuclear aggregation of β‐arrestin‐1 may be attributed to its location in the MIB1 binding motif, which prevented specific binding of β‐arrestin‐1 to MIB1 by adding O‐GlcNAc group. Our study elucidates the PTM‐associated molecular mechanism underlying β‐arrestin‐1 stability in T‐2 toxin‐exposed glomerular podocytes in response to glucose metabolism.

Because our study proved that β‐arrestin‐1 O‐GlcNAcylation was the starting point for the T‐2 toxin‐induced podocyte damage, we proposed that inhibition of OGT may be a valid method for alleviation of podocyte injury. Clinical and experimental studies have indicated that OGT is an ideal intervention target for the therapy of multiple metabolic‐related diseases. Downregulation of OGT through pharmacological or genetic interventions is sufficient to prevent proliferation of human gastric tumor cells, NUGC‐3, and HEK293 in vitro, and relieve renal dysfunction and podocyte shedding in diabetic mice and patients with diabetic nephropathy.^[^ ^37^ ^]^ In the current study, our results showed that insufficient OGT alleviated glomerular abnormalities and proteinuria in T‐2 toxin‐stimulated mice. Additionally, OGT deletion repressed the ubiquitin‐dependent proteolysis of β‐arrestin‐1, lessened β‐arrestin‐1 nuclear aggregation and its interaction with p300, inhibited H4K16 acetylation and p‐mROT (s2448) protein levels as well as ameliorated podocyte damage and autophagy in T‐2 toxin‐fed mice. Thus, the present research confirms that OGT intervention is a feasible approach for the alleviation of podocyte injury in T‐2 toxin‐fed model mice, and it expands our current comprehension of the treatment of podocyte injury‐associated glomerulonephropathy.

The limitation of this study is that the function of rAAV9‐mediated knockout of β‐arrestin‐1 in T2 toxin‐fed mice was validated at the tissue level but not at the cell level. Where β‐arrestin‐1 functions may not be limited to only podocytes. For example, since podocytes and mesangial cells are structurally and functionally connected, β‐arrestin‐1 knockdown in either podocytes or mesangial cells may cause glomerular podocyte injury. It is a very complex process and needs quite much time to explore, and our future study will focus on it.

## 4 Conclusion

We revealed the mechanisms of metabolic reprogramming and podocyte injury in response to T‐2 toxin infection. T‐2 toxin exposure upregulates GLUT1 expression and promotes β‐arrestin‐1 O‐GlcNAcylation both in vitro and in vivo. Hyper‐O‐GlcNAcylation of β‐arrestin‐1 can enhance histone acetylation and inhibit autophagy in T‐2 toxin‐resistant glomerular podocytes. Mechanistically, OGT‐mediated O‐GlcNAcylation of β‐arrestin‐1 at Thr98 stabilizes β‐arrestin‐1 to suppress autophagy via activating the mTOR pathway through recruitment of p300 to upregulate H4K16 acetylation. This study broadens our comprehension of the molecular mechanisms in regulating β‐arrestin‐1 generation and offers new insight into the significance of protein O‐GlcNAcylation in T‐2 toxin infection‐induced podocyte injury.

## 5 Experimental Section

### Animals and Treatment

Male C57BL/6N mice (5 weeks old) from the Beijing Vital River Laboratory Animal Technology Co., Ltd (production license: SCXK 2022–0013) were acclimatized to environment in a dark‐light cycle of 12 h/12 h for a week. Thirty mice were separated into two experimental groups: the T‐2 toxin‐fed group (*n =* 15) received 2 mg k^−1^g T‐2 toxin by gavage daily for 4 weeks, while the normal group (*n =* 15) adopted a standard diet. At the 4th week of T‐2 toxin feeding, 24 h urine of each mouse was gathered in a metabolic cage and stored at −80 °C for follow‐up experiments.

### Generation of Kidney‐Specific OGT Knockout Mice

A combination of rAAV9 and renal vein injection was optimal for kidney‐targeted gene delivery.^[^ ^38^ ^]^ For knockdown of β‐arrestin‐1 and OGT, mice aged 4–5 weeks with C57BL/6N background were administered with rAAV9 via retrograde renal vein injection. A detailed operation process was implemented as previously reported.^[^ ^38^ ^]^ In brief, after anesthetization, the kidneys were exposed through the flank incision, and rAAV9 particles were injected through retrograde renal vein injection with a 31G needle. After injection of rAAV9 for 15 min, the clamp was removed, and the incision was sutured. Following two weeks of injection, epifluorescence microscopy and Western blot analysis were conducted to verify the knockout efficiency of rAAV9 in mouse kidneys.

### Reagents

T‐2 toxin was provided by Shanghai Yuanye Biotechnology Co., Ltd. (Shanghai, China). MG‐132 was acquired from MCE Corporation (Shanghai, China). RPMI‐1640 medium, fetal bovine serum (FBS) along with opti‐MEM medium were acquired from Excell Bio Corporation (Wellington, New Zealand). BCA Protein Assay Kit, albumin enzyme‐linked immunosorbent assay (ELISA) kit along with the assay kits of creatinine and urine protein were purchased from Nanjing Jiancheng Bioengineering Institute (Nanjing, China). Eosin dye, periodic acid, cell lysis RIPA buffer, Kit of nuclear protein extraction, phenylmethanesulfonyl fluoride (PMSF) along with 4, 6‐diamidino‐2‐phenylindole (DAPI) staining solution were provided by Sigma‐Aldrich Inc. (St. Louis, MO, USA). Alexa Fluor 488 goat anti‐rabbit IgG (A11008), TRIzol reagent along with iTaq Universal SYBR Green Supermix were purchased from Bio‐Rad Inc (California, USA). A reverse transcription system kit, dNTPs as well as RNase inhibitors were acquired from Vazyme Biotechnology Co., Ltd (Nanjing, China). HRP‐conjugated mouse anti‐IgG (HAF007) and HRP‐conjugated goat anti‐IgG (HAF017) were purchased from Proteintech Group, Inc. (Chicago, USA). Lipofectamine 2000, TRIzol reagent, and were acquired from Invitrogen Biotechnology Co., Ltd (Shanghai, China). UDP‐GlcNAc, GlcNAc, N‐acetyl‐d‐glucosamine 6‐phosphate (GlcNAc‐6‐p), and Glucose ELISA assay kit were provided by Shanghai Ruifan Biological Technology Co, Ltd (Shanghai, China). Rabbit anti‐Histone H3 (ab1791), rabbit anti‐Histone H4 (ab10158), rabbit anti‐ubiquitin (ab7780), anti‐GLUT1 (ab115730), anti‐β‐arrestin‐1 (ab31868), anti‐β‐arrestin‐2 (ab206972), anti‐Nephrin (ab216341), anti‐Pdocin (ab181143), anti‐p300 (ab275378), rabbit anti‐H4K16ac (ab109463), anti‐p‐mTOR (Ser2448, ab109268), anti‐mTOR (ab134903), anti‐p‐mTOR (Ser2481, ab232486), rabbit anti‐LC3 (ab192890), anti‐p62 (ab109012), anti‐Beclin 1 (ab207612) and Mouse anti‐synaptopodin (MAB4919) were obtained from Abnova Corporation (Taipei, Taiwan). Anti‐ATG5 (ab108327), anti‐MIB1 (ab124929), and mouse anti‐Lamin A (ab8980) were acquired from Abcam Corporation (Cambridge, MA, USA). Mouse anti‐O‐GlcNAc (Sc‐59623) and mouse anti‐β‐actin (Sc‐8432) were acquired from Santa Cruz Biotechnology Co., Ltd (Santa Cruz, USA). Rabbit HRP‐conjugated anti‐IgG (#AP132P) and rabbit anti‐OGT (#24083) were acquired from Cell Signaling Technology (Cambridge, USA). Mouse anti‐acylation antibody (PTM‐6680) was acquired from Hangzhou Jingjie Biotechnology Co., Ltd (Hangzhou, China).

### Blood and Tissue Sample Preparation

Pentobarbital sodium was used to anesthetize mice at dose of 20 mg k^−1^g. After collection of inferior vena cava blood samples, the blood was centrifuged to acquire serum samples and then stored at −80 °C. Following serum sample collection, the renal cortex was isolated on ice. Next, kidney cortex tissues were sliced into pieces for glomeruli separation, histology examination, electron microscopy analyses, and total RNA isolation along with Western blot assay. A graded sieving technique was adopted to isolate glomeruli with sizes of 250, 150, and 75 µm as reported.^[^ ^15a^ ^]^

### Biochemical and Histologic Analysis

Serum creatinine, urea nitrogen, uric acid, urinary albumin, as well as urinary creatinine levels were measured using commercially available biochemical kits. Kidney cortex tissues were taken to fix in 4% paraformaldehyde, embedded in paraffin, and cut into 4 µm sections. After deparaffinization, kidney tissue sections were taken for HE or PAS staining. Finally, three randomly selected glomeruli images were observed with a light microscope (Olympus BX51, Tokyo, Japan).

### TEM

Electron microscopic sample processing and testing were performed^[^ ^15a^ ^]^ in the electron microscopic core lab of Nanjing Medical University. The thickness of the peripheral GBM and foot process width were calculated by the ImageJ software (National Institutes of Health, USA).

### RNA‐seq Analysis

Thirty mg glomerular tissue was used to isolate total RNA with the Ultrapure RNA Kit (CWBIO, CW0581M). Briefly, after trituration in 1 mL TRIzol, the homogenized tissue was added to chloroform for incubation for 5 min and shaken vigorously. After centrifugation, the upper water phase was moved into an adsorption column and then eluted with RNase‐free water. PCR amplification was performed to construct a cDNA library. RNA‐seq was done with the PE150 sequencing strategy on the Illumina second‐generation high‐throughput sequencing platform. Poor quality reads were filtered, while clean reads data were processed by Tophat2 and Cufflinks software to complete transcriptome comparison and fragment splicing analysis.

### Cell Culture and Treatment

Conditionally immortalized heat‐sensitive human podocyte cell line was purchased from Beijing Future Biotech Co. Ltd (Beijing, China) and was cultured in RPMI 1640 medium including 10% FBS and 1% penicillin‐streptomycin solution at 37°C, 5% CO_2._ The concentration of T‐2 toxin was 40 nM according to a previous study.^[^ ^39^ ^]^ After 24 h exposure to T‐2 toxin, metabolites and related protein and gene expression in human podocytes were measured. siRNAs were transfected into podocytes to knock down β‐arrestin‐1, p300, and MIB1 in the presence or absence of T‐2 toxin. Overexpression of p300 by a p300‐rAAV9 transfection was adopted in this study.

### Immunofluorescence Staining and Confocal Microscopy

Immunofluorescence staining was conducted to detect GLUT1, β‐arrestin‐1, β‐arrestin‐2, Nephrin, and Podocin in podocytes and mouse glomerulus. Specific signals were observed by means of Alexa Fluor 488 secondary antibody (Invitrogen). The cell nucleus was stained with DAPI (ab285390, Abcam), followed by observation using a laser scanning confocal microscope (ZEISS, Germany) system. To monitor autophagosome formation in conditions of podocytes, the tandem GFP‐RFP‐LC3 adenovirus construct (Shanghai Zorun Biology, China) was adopted in this study.

### Co‐IP

Co‐IP was performed to measure the interaction of β‐arrestin‐1 with p300, OGT, O‐GlcNAc, H4K16ac, MIB1, and ubiquitin in podocytes. Among them, lentiviruses containing Flag‐MIB1 and HA‐β‐arrestin‐1 or its mutants were synthesized and constructed by Shanghai Jikai Biotechnology Co., Ltd (Shanghai, China). The assay was performed as previously described.^[^ ^40^ ^]^ In brief, after lysis in IP lysis buffer as well as sonication on ice, the cell lysate was precipitated by centrifugation. For IP, cell lysate was treated with β‐arrestin‐1 or H4K16ac antibody with rotation followed by treatment with protein A/G‐conjugated agarose beads. After washing and elution, beads‐bound proteins were analyzed by Western blot.

### ChIP‐seq Analysis

ChIP‐seq analysis was performed for the analysis of H4K16ac binding DNA fragments as previously described.^[^ ^41^ ^]^ Briefly, after washing, cross–link, quenching, trituration, and centrifugation, the cell precipitate was incubated with 1 mL lysis buffer. Following centrifugation and washing, 1 µL MNase (NEB, M0247S) was added into the digestion buffer and incubated, and then quenched with EDTA. The obtained IP was incubated with sheared chromatin, antibody, 5 Protein G beads, and dilution buffer overnight at 4 °C. After washing and elution, the DNA fragment was obtained using a DNA Extraction Kit (Cell Signaling Technology) and subsequently purified with a DNA purification kit (TIANGEN, #DP214‐03).

### Construction of β‐arrestin‐1 Knockout Human Podocyte Line

β‐arrestin‐1 knockout podocyte was established with a Crispr‐Cas9 system as described.^[^ ^42^ ^]^ pLenti‐CRISPR‐V2 (add gene 52961, puromycin resistant) was modified to acquire resistance to neomycin. Oligos corresponding with gRNA were designed, annealed, as well as ligated into BsmBI‐cut pLenti‐CRISPR‐V2 vectors as instructed by the vendor. Cells were infected with lentiviruses coding *β‐arrestin‐1*‐sgRNA (neomycin resistant) and then selected with G418 (MCE HY‐17561). The details of Lentiviral transfection were described previously.^[^ ^43^ ^]^ Crispr‐Cas9 plasmid cuts β‐arrestin‐1 genomic loci between aa123 and aa243. The knockout efficiency of Crispr‐Cas9 in podocytes was evaluated by measuring the mRNA and protein of β‐arrestin‐1 through quantitative real‐time PCR (qRT‐PCR) and Western blot.

### Metabolites Analysis in Fructose‐Exposed Podocytes

To extract metabolites from quenched podocyte culture supernatants, each sample was mixed with chilled methanol: acetonitrile (2:2, v/v), swirled three times for 1 min each time and incubated for 5 min. Next, the mixture was added with chilled HPLC‐certified water and centrifuged. Finally, the supernatant of the podocyte sample was transferred into a new tube for implementing UHPLC‐QTOF‐MS analysis in Suzhou Mass‐Elife Biotechnologies Co., Ltd (Suzhou, China). UDP‐GlcNAc, GlcNAc, GlcNAc‐6‐p, and glucose were quantified using ELISA kits. The data acquisition and heatmap analysis were performed by Suzhou Mass‐Elife Biotechnologies Co., Ltd (Suzhou, China).

### IP Assay Coupled with MS

Podocyte lysates were taken for incubation with anti‐O‐GlcNAc and anti‐β‐arrestin‐1 antibodies at 4 °C overnight, and then added with protein A/G agarose beads. Coomassie blue was used for staining and separation of immune complexes. Stained protein bands were subjected to identify potential O‐GlcNAc‐modified or β‐arrestin‐1 binding proteins by Suzhou Mass‐Elife Biotechnologies Co., Ltd (Suzhou, China). After dissolution in chilled methanol: acetonitrile: H_2_O (2:2:1, v/v/v), sonication, and centrifugation, the supernatant was dried in a vacuum centrifuge and then re‐dissolved in acetonitrile: water (1:1, v/v) for LC‐MS analysis.

### Histone H4 Acetylation and β‐arrestin‐1 O‐GlcNAcylation Site Mapping in T‐2 Toxin‐Exposed Podocytes

MS strategy was employed to identify Histone H4 acetylation and β‐arrestin‐1 O‐GlcNAcylation sites as reported.^[^ ^44^ ^]^ Briefly, immunoprecipitated Histone H4 or β‐arrestin‐1 from podocytes underwent SDS‐PAGE. The band corresponding to Histone H4 or β‐arrestin‐1 was separated, reduced with DTT, and digested with trypsin. Prepared samples were then subjected to LC‐MS/MS analysis. Raw data files were processed using Byonic v2.13.17 (Protein Metrics, California, USA). Peak lists were searched in the human Uniprot database using Sequest. A database of 70 O‐glycan modifications was also searched and used automated scoring to remove low‐confidence peptides. The Percolator node was performed to determine false discovery rates (FDRs). A 5% peptide FDR was used to filter results. Byonic searching result was further processed using Byologic (Protein Metrics).

### qRT‐PCR Analysis

Total RNA was isolated by TRIzol reagent (Invitrogen). qRT‐PCR was performed using iTaq Universal SYBR Green Supermix. The mRNA expression was calculated following a standard delta‐delta Ct method. The sequences of specific primers (5′→3′) were listed as follows: mouse ARRB1 (β‐arrestin 1), F, ACATCGGGAAGTTCCAGAG, R, AGTCCTCAAATACAATGTCGTC; mouse ARRB2 (β‐arrestin 2), F, ACAAAGAGCTGTACTACCAC, R, ATCTTCTTGACGGTCTTGG; mouse ACTB (β‐actin), F, GCTATGTTGCTCTAGACTTCG, R, GGATTCCATACCCAAGAAGG; human ARRB1, F, CTTTGTGGACCACATCGAC, R, TCACATAGACTCTCCGCTC; human ARRB2, F, AGGATACAGGAAAGGCCTG, R, TCAGACATGAGGAAGTGGC; human ACTB, F, GAAGATCAAGATCATTGCTCCTC, R, ATCCACATCTGCTGGAAGG. All samples were run in three replicates.

### Western Blot Analysis

RIPA buffer was performed to lyse tissue or cells, and the samples were isolated by SDS‐PAGE, and transferred to PVDF membrane, followed by incubation with primary antibody after blocking. After treating with HRP‐conjugated secondary antibodies, the Enhanced Chemiluminescent reagent (Thermo Scientific) was performed for band detection. Primary antibody concentrations were as follows: anti‐Nephrin (1:2000), anti‐Podocin (1:2000), anti‐ubiquitin (1:1000), anti‐GLUT1 (1:2000), anti‐β‐arrestin‐1 (1:1000), anti‐β‐arrestin‐2 (1:1000), anti‐O‐GlcNAc (1:1000), anti‐Lamin A (1:1000), anti‐IgG (1:1000), anti‐β‐actin (1:2000), anti‐OGT (dilution 1:2000), anti‐Histone H3 (1:1500), anti‐Histone H4 (1:1500), anti‐p300 (1:1500), anti‐H4K16ac (1:1500), anti‐p‐mTOR (Ser2448, 1:1500), anti‐p‐mTOR (Ser2481, 1:1500), anti‐LC3 (1:1500), anti‐p62 (1:10 000), anti‐Beclin 1 (1:1000), anti‐ATG5 (1:3000), and anti‐MIB1 (1:1500).

### Quantitation and Statistical Analysis

Data were exhibited as mean ± SEM and analyzed by the GraphPad Prism software (Version 5.0). Student's unpaired *t*‐test was conducted to calculate two‐tailed p values between two groups. One‐way Analysis of Variance (ANOVA) was performed for multiple comparisons among three or more groups. Tukey's post hoc test was used to compare each average to other averages, and Dunnett's post hoc test was used to compare each average to the same control average. p values were denoted as follows: * *p <* 0.05, ** *p <* 0.01, and *** *p <* 0.001.

### Ethics Approval Statement

All experimental procedures were performed according to the regulations of the Institutional Animal Care and Use Committee of Jiangnan University. All efforts were made to minimize animal suffering and to reduce the number of animals used.

## Conflict of Interest

The authors declare no conflict of interest.

## Author Contributions

T.L., J.Z., and Y.C. conceived the study and designed the experiments; T.L. performed the experiments with the help of J.Z., W.S., C.H., and T.C.; J.Z., C.H. and T.C. helped with the animal experiments; J.Z., W.S., and S.Z. processed and analyzed the experimental data with constructive discussions; T.L. wrote the manuscript; J.Z. and Y.C. edited the manuscript; J.Z., W.S., and Y.C. supervised the study. All authors have read and approved the final manuscript.

## Supporting information

Supporting Information

## Data Availability

The data that support the findings of this study are available from the corresponding author upon reasonable request.
