# Supplementary material for: T‐2 Toxin‐Mediated β‐Arrestin‐1 O‐GlcNAcylation Exacerbates Glomerular Podocyte Injury via Regulating Histone Acetylation
Source: Adv Sci (Weinh). 2023 Dec 11;11(7):2307648. doi: 10.1002/advs.202307648 (PMC10870076; doi:10.1002/advs.202307648)
Supplement: Supplementary file 1 — Supporting Information [file ADVS-11-2307648-s001.pdf]

ADVANCED SCIENCE
Open Access

Supporting Information

for *Adv. Sci.*, DOI 10.1002/advs.202307648

T-2 Toxin-Mediated *β*-Arrestin-1 O-GlcNAcylation Exacerbates Glomerular Podocyte Injury via Regulating Histone Acetylation

Tushuai Li, Wenxue Sun, Shenglong Zhu, Chengsheng He, Tong Chang, Jie Zhang and Yongquan Chen**

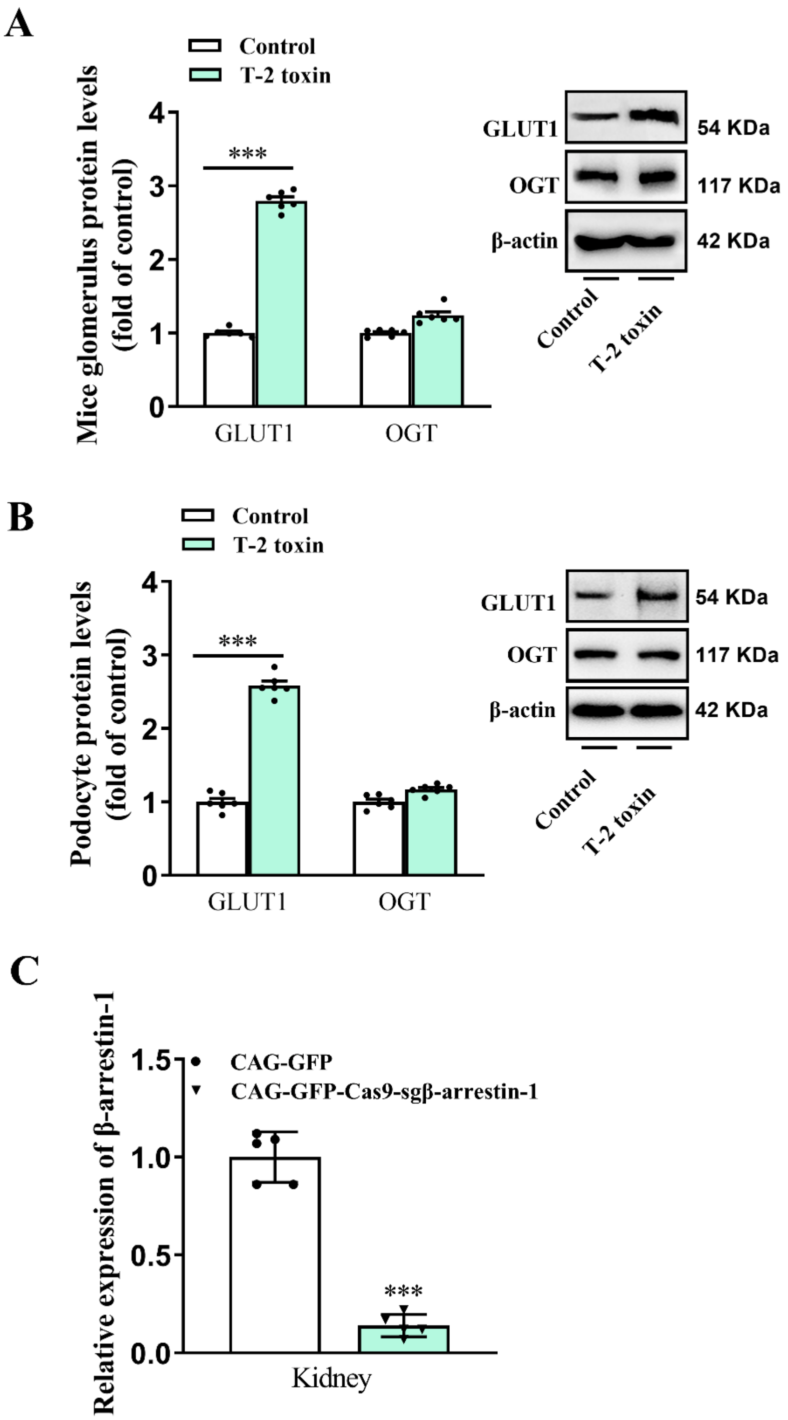

Supplementary Figure 1. The GLUT1 and OGT protein levels and β-arrestin-1 mRNA levels (A-B) Western blot analysis of GLUT1 and OGT protein levels in mouse glomeruli (A) and *in vitro* podocytes (B) (n = 6). Relative protein levels of GLUT1 and OGT were normalized to β-actin. (C) Normalized β-arrestin-1 mRNA expression in kidneys of mice receiving rAAV9 injection by renal vein injection was revealed by PCR method. Quantitative data are presented as mean ± S.E.M. $^{*}p < 0.05$, $^{**}p < 0.01$, and $^{***}p < 0.001$, unpaired *t* test was used.

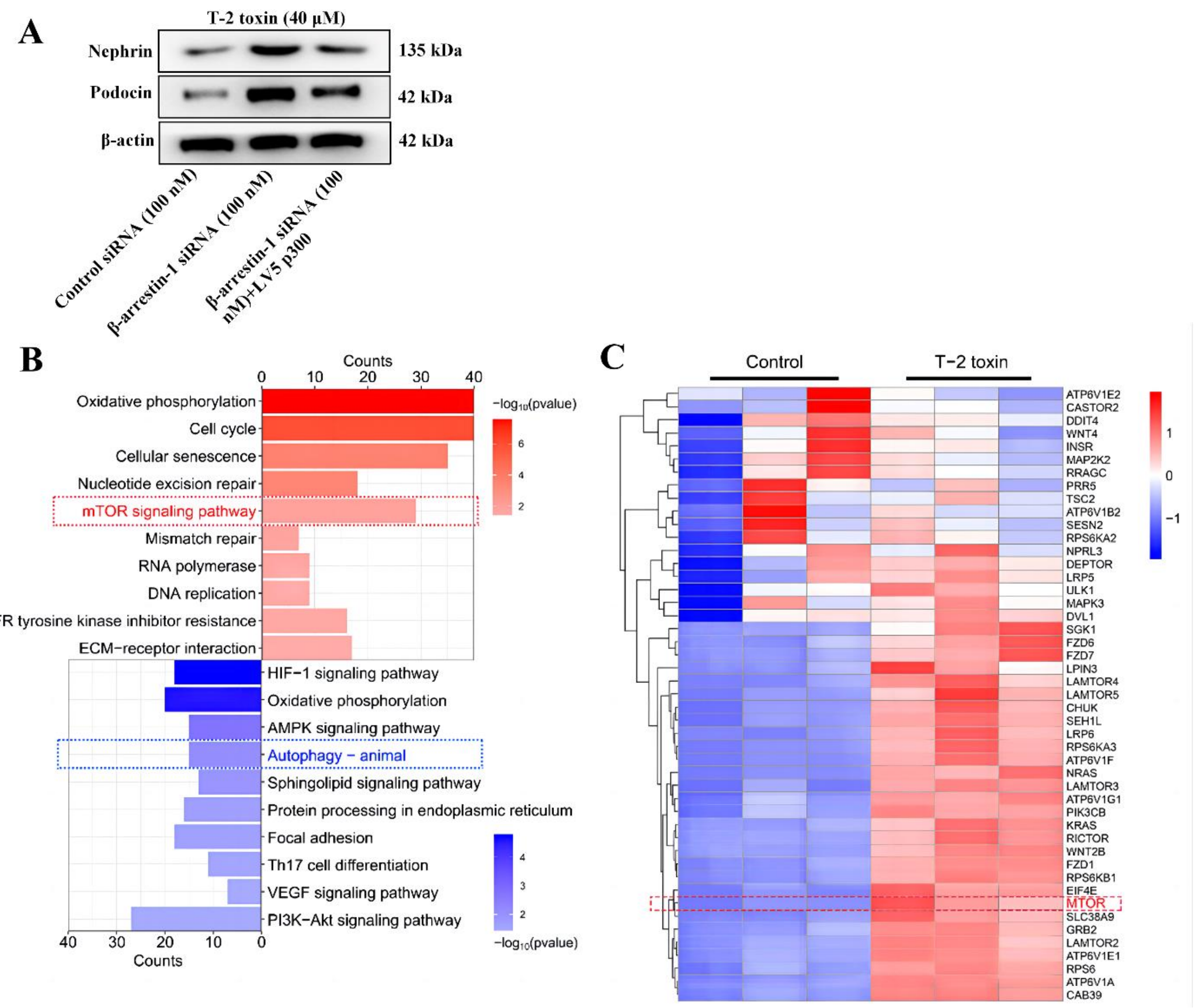

Supplementary Figure 2. RNA-Seq analysis of signaling pathways and differential dene changes

(A) Western blotting of Nephrin and Podocin proteins under the treatment of p300 siRNA and β-arrestin-1 siRNA. (B) KEGG pathway analysis of RNA-Seq results showed significantly enriched signaling pathways in T-2 toxin-exposed podocytes vs its normal controls. (C) RNA-Seq heatmaps showed significant changes of mRNAs in T-2 toxin-exposed podocytes vs its normal controls.

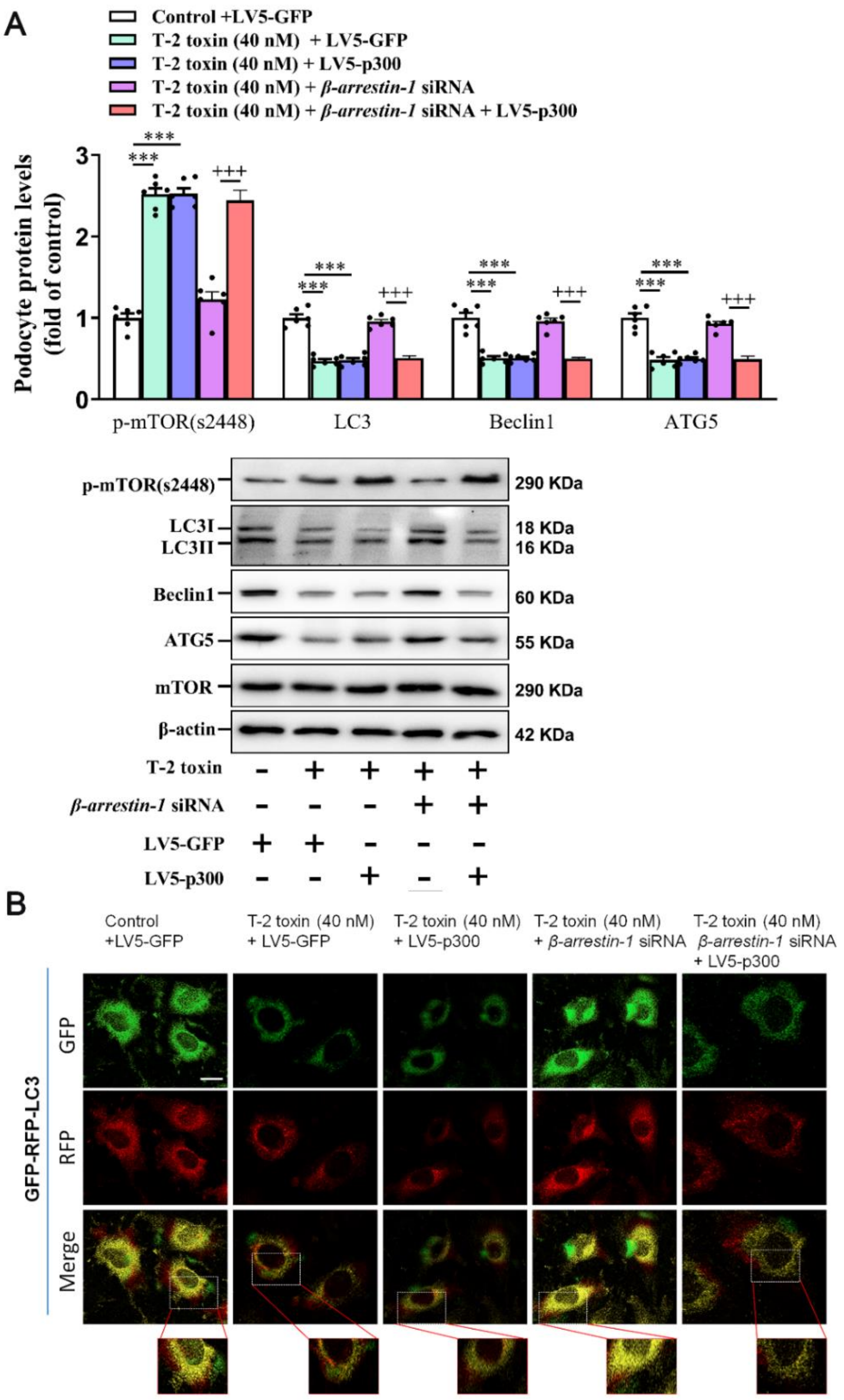

Supplementary Figure 3. T-2 toxin enhances the interaction of β-arrestin-1 and p300 to activate the mTOR pathway and inhibit autophagy in podocytes

(A) Western blot analysis of p-mTOR (s2448), LC3, Beclin1, and ATG5 in podocytes cultured with or without T-2 toxin in the presence or absence of LV5-p300 and *β-arrestin-1* siRNA (n = 6). (B) Representative images of LC3 staining by measurement of fluorescent intensity in podocytes (n = 3). Scale bar, 20 μm. Relative protein levels of p-mTOR (s2448), LC3, Beclin1, and ATG5 were normalized to mTOR and β-actin, respectively. Quantitative data are presented as mean ± S.E.M. $^{*}p < 0.05$, $^{**}p < 0.01$, $^{***}p < 0.001$; $^{+}p < 0.05$, $^{++}p < 0.01$, $^{+++}p < 0.001$; one-way ANOVA followed by Tukey's post hoc test was used.

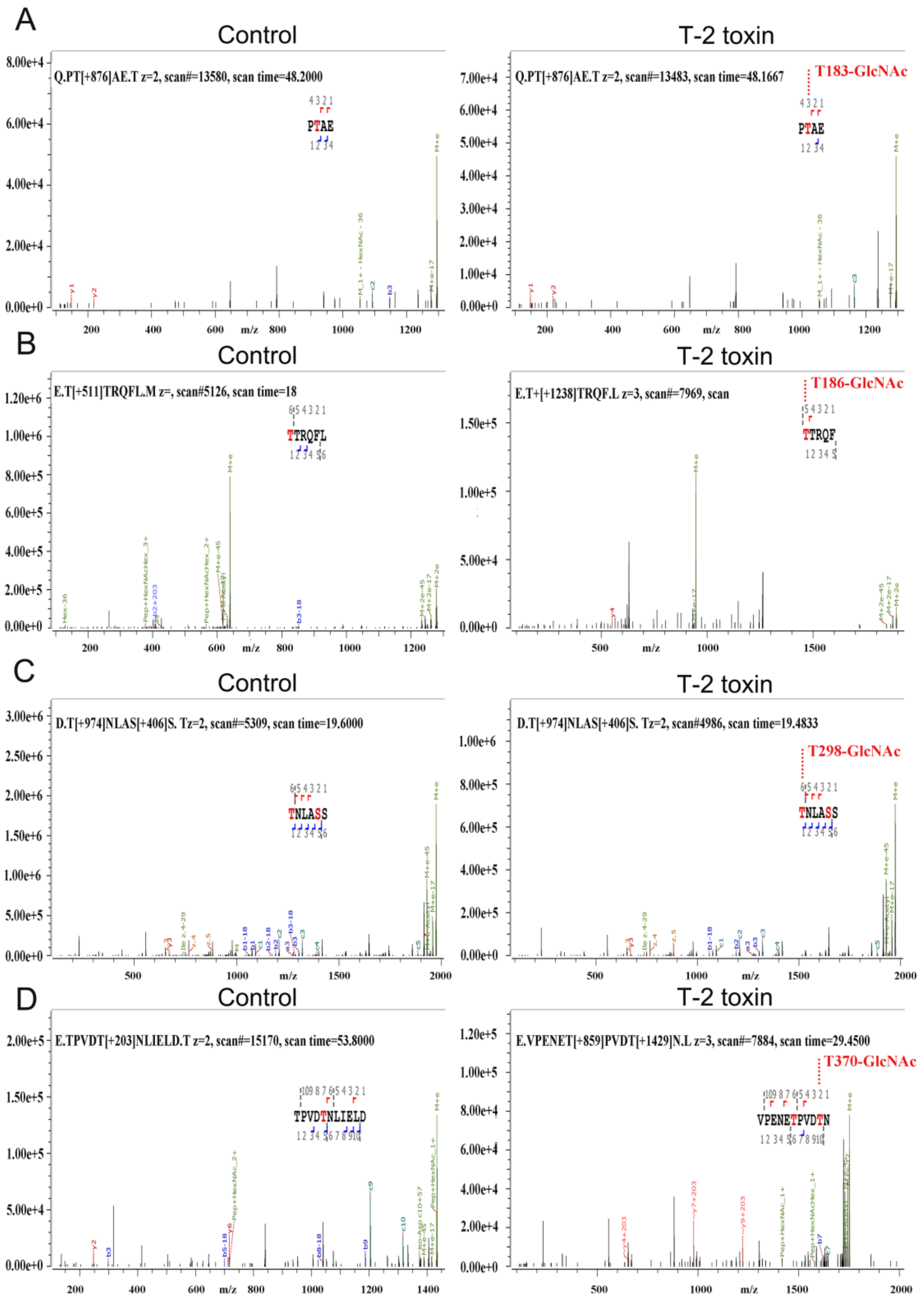

Supplementary Figure 4. The GlcNAc-modified amino acid site of β-arrestin-1 in podocytes with or without T-2 toxin exposed.

(A-D) The LC-MS/MS analysis results of other four peptides significantly modified by GlcNAc and their corresponding unmodified peptides from a tryptic digest of β-arrestin-1 in podocytes with or without T-2 toxin exposed.

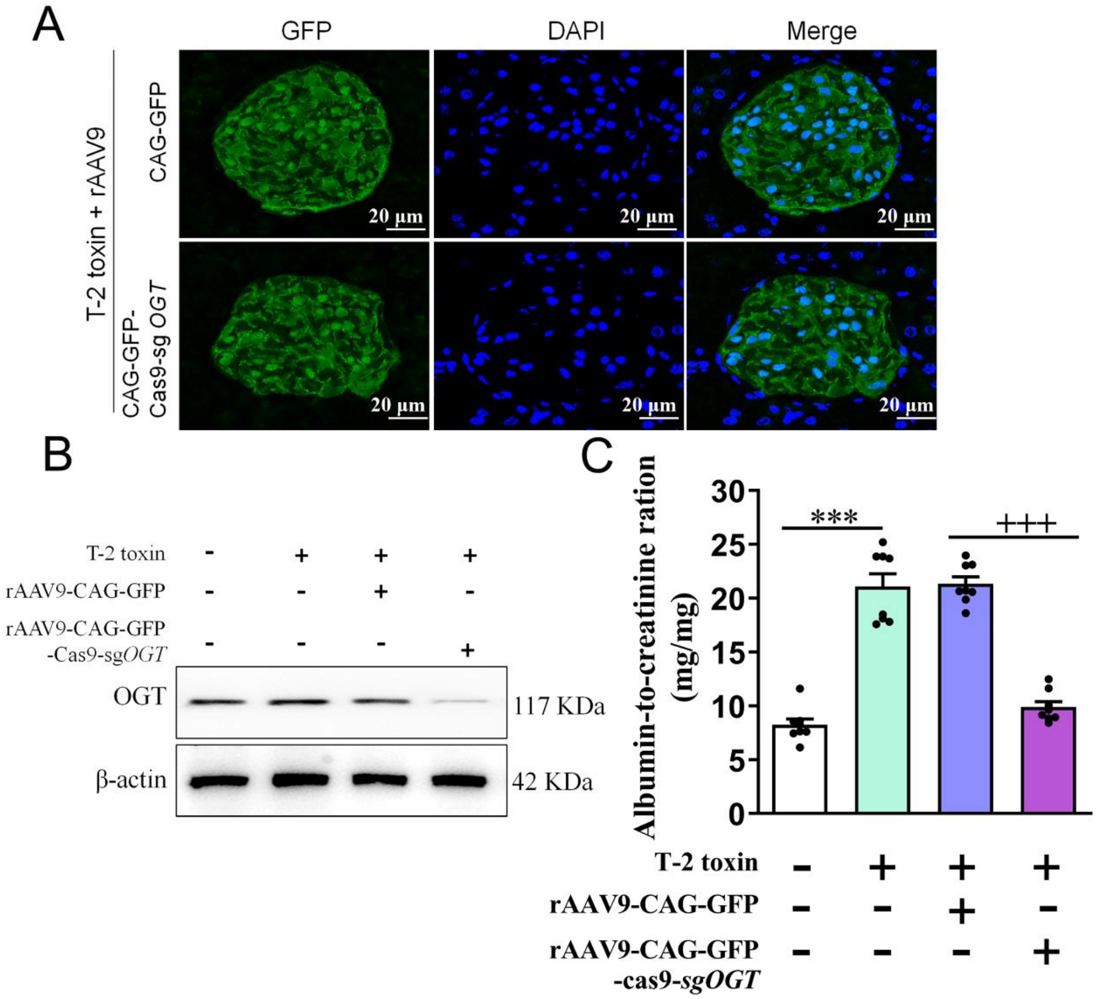

Supplementary Figure 5. The transfection efficiency of rAAV9 in mouse kidney and urine albumin-to-creatinine ratio of OGT knockout mice

(A) The transfection efficiency of CAG-GFP-Cas9-sg*OGT* or CAG-GFP in mouse glomeruli detected by immunofluorescence staining (n = 4, scale bar, 20 μm). (B) OGT protein levels were detected using Western blot analysis in isolated glomeruli from T-2 toxin-fed mice infected with CAG-GFP-Cas9-sg*OGT* or CAG-GFP (n = 3). (C) The urine albumin-to-creatinine ratio in T-2 toxin-fed mice transfected with CAG-GFP-Cas9-sg*OGT* or CAG-GFP (n = 6). Quantitative data are presented as mean ± S.E.M. $^{*}p < 0.05$, $^{**}p < 0.01$, $^{***}p < 0.001$; $^{+}p < 0.05$, $^{++}p < 0.01$, $^{+++}p < 0.001$; one-way ANOVA followed by Tukey's post hoc test was used.

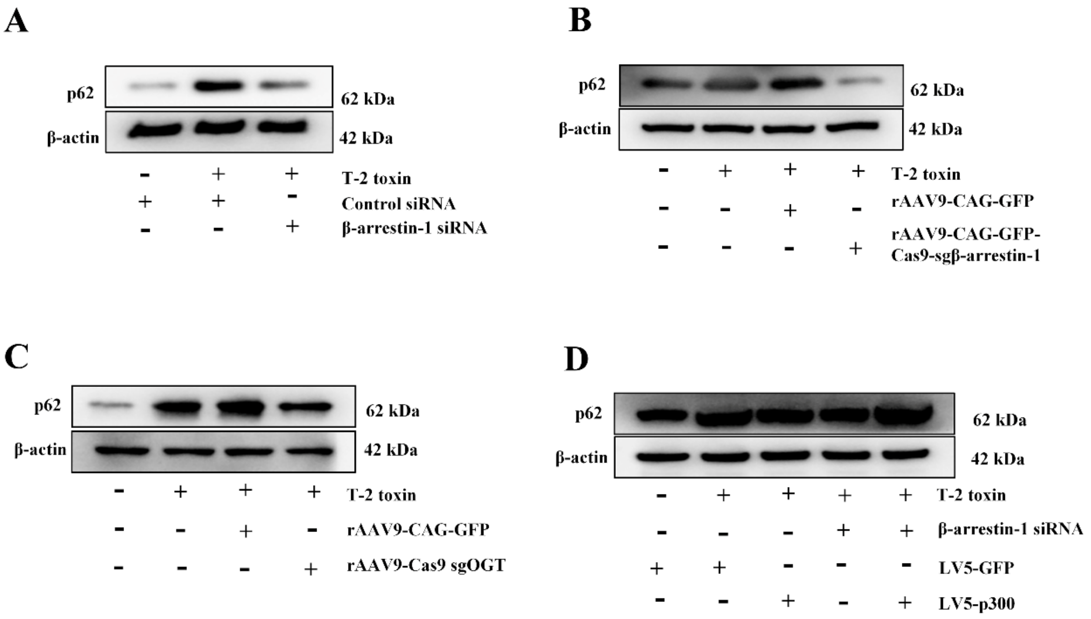

Supplementary Figure 6. β-arrestin-1 mediates p62 protein levels

(A) Western blotting of p62 in podocytes treated with T-2 toxin and β-arrestin-1 siRNA. (B-C) Western blotting of p62 in isolated glomeruli from T-2 toxin-fed mice infected with rAAV9 CAG-GFP-Cas9-sgβ-arrestin-1 or rAAV9 CAG-GFP-Cas9-sgOGT. (D) Western blot of p62 in T-2 toxin-treated podocytes in the presence or absence of LV5-p300 and *β-arrestin-1* siRNA.
